# The IL-33/ST2 Axis Promotes Traumatic Heterotopic Ossification by Driving Macrophage and Mast Cell-Mediated Inflammation via Autophagy Defects

**DOI:** 10.7150/ijbs.122706

**Published:** 2026-01-01

**Authors:** Zhenyu Chen, Yi Xu, Cheng Qu, Gang Luo, Zhuochang Cai, Hang Liu, Ziyang Sun, Chao Zhou, Juehong Li, Cunyi Fan

**Affiliations:** 1 Department of Orthopedics, Shanghai Sixth People's Hospital Affiliated to Shanghai Jiao Tong University School of Medicine, Shanghai, 200233, P. R. China.; 2 Shanghai Engineering Research Center for Orthopaedic Material Innovation and Tissue Regeneration, Shanghai, 201306, P. R. China.

**Keywords:** IL-33/ST2 axis, Autophagy, Heterotopic ossification

## Abstract

Trauma-induced Heterotopic ossification (tHO) is the abnormal osteogenesis occurring in soft tissues after traumatic musculoskeletal injury, which can lead to severe limb movement impairment or even disability. Recent studies have indicated that macrophages and mast cells play a crucial role in tHO, although their precise activation mechanism remained to be elusive. Here, we unveil a novel mechanism in which interleukin-33 (IL-33)—an alarmin in the mammalian innate immune response to trauma—rapidly increases upon tendon injury and binds to its receptor ST2 (IL-1RL1) on macrophages and mast cells. This binding initiates M2 polarization in macrophages and degranulation in mast cells, thereby promoting osteogenic differentiation during tHO formation. Mechanistically, the IL-33/ST2 axis leads to the autophagy defection in macrophages and mast cells. ST2-knockout (ST2-/-) markedly restores autophagy and mitigates tHO. Furthermore, we identified activation of the PI3K/AKT/mTOR pathway as a critical mechanism mediating IL-33-induced autophagy suppression. Restoration of autophagy via PI3K/AKT/mTOR pathway inhibitors similarly counteracts the aberrant osteogenic healing effects induced by IL-33. To explore a therapeutic strategy, we fabricated a bacterial cellulose (BC) hydrogel composite scaffold loaded with soluble ST2 (sST2), based on a competitive inhibition approach. These scaffolds successfully sequestered IL-33 during the early inflammatory phase, thereby alleviating macrophage- and mast cell-mediated inflammation and tHO formation. By identifying overexpression of the IL-33/ST2 axis in human HO tissues and further validating through animal experiments, this study elucidates how the alarmin IL-33 contributes to tHO via immune regulation. Our findings reinforce the pivotal role of autophagy in attenuating HO and provide new translational perspectives for its clinical treatment.

## Introduction

Heterotopic ossification (HO) is defined as the aberrant formation of bone within soft tissues and could be categorized into congenital and acquired forms. Trauma-induced heterotopic ossification (tHO), the most prevalent acquired form, commonly arises as a complication following severe burns, fractures, spinal cord injuries, and extensive orthopedic procedures, such as total hip arthroplasty and elbow release surgery 1-3. This condition can lead to serious complications including nerve entrapment, pressure ulcers, restricted joint mobility, and permanent disability 3-5. The prevention of tHO is especially crucial in patients with high-risk factors, including extensive surgical exposure, soft tissue trauma, obesity, and prolonged immobilization 6. Despite being commonly used, nonsteroidal anti-inflammatory drugs (NSAIDs) and radiotherapy show limited efficacy 7. Further exploration of the underlying mechanisms is needed to provide new insights into the prevention of HO.

Current evidence suggests that heterotopic ossification develops through a complex pathological process involving the recruitment and activation of immune cells, the induction of osteogenic signaling pathways such as bone morphogenetic proteins (BMPs) and inflammatory cytokines, and the subsequent formation and maturation of ectopic bone tissue 8. During the first step, the activation of mast cells and macrophages plays a significant role 9-10. On one hand, macrophages are highly plastic immune cells that can polarize into distinct functional phenotypes—M1 (classically activated) and M2 (alternatively activated)—in response to various environmental cues 11. Notably, M2 macrophages contribute to the formation of heterotopic ossification by secreting a variety of pro-osteogenic factors, including TGF-β, IL-10, VEGFA, and BMP-2, thereby creating a microenvironment conducive to ectopic bone formation 12. On the other hand, mast cells have been implicated in the early stages of heterotopic ossification by contributing to a pro-inflammatory and pro-angiogenic microenvironment. Upon activation, mast cells release a wide array of mediators through degranulation, including IL-6, MCP-1, TNF-α, and VEGF, which facilitate immune cell recruitment, vascular remodeling, and the activation of osteogenic pathways 9,13. During osteogenesis, tendon-derived stem cells (TDSCs) function as a critical bridging element. Post-traumatic inflammatory activation generates a distinctive immune microenvironment through the release of diverse cytokines and chemokines, which drives TDSCs toward aberrant differentiation—where physiological tendon healing is superseded by pathological osteogenesis, ultimately culminating in heterotopic bone formation 14. Therefore, modulating M2 macrophage polarization and mast cell degranulation could potentially reshape the formation of traumatic heterotopic ossification (tHO) by affecting TDSCs.

Interleukin-33 (IL-33) is a tissue-derived nuclear cytokine belonging to the IL-1 superfamily, with diverse biological functions. It is typically expressed by fibroblasts, endothelial cells, smooth muscle cells, and other cell types. Following tissue injury, IL-33 surges within a short period and functions as an alarmin to activate cells expressing the ST2 (IL-1RL1) receptor 15-16. Recent studies indicate that IL-33-induced immune cell activation is involved in various pathophysiological processes following trauma. Xu et al. demonstrated that IL-33 mediates the recruitment and activation of eosinophils in mice during acute liver injury 17. He et al. found that IL-33 promotes M2 polarization of macrophages while enhancing neo-angiogenesis and extracellular matrix deposition, which can accelerate wound healing in diabetic mice 18. The IL-33/ST2 axis is critically involved in immune modulation and the maintenance of cellular homeostasis, facilitating wound healing and tissue regeneration, a role that has been extensively acknowledged in the literature. Nevertheless, no studies have yet discussed the effect of the IL-33/ST2 axis on post-traumatic heterotopic ossification.

Autophagy is a highly regulated, dynamic cellular process orchestrated by eukaryotic cells, constituting an adaptive mechanism to maintain cellular homeostasis and respond to microenvironmental perturbations under conditions such as cellular injury, oxidative stress, or metabolic demand 19. Literature reports suggest that dysfunction of autophagy-lysosome pathways contributes to ectopic bone formation, while IL-33/ST2 axis has been shown to inhibit autophagy and exert neuroprotective effects 20-22. Accordingly, we propose that IL-33 may play a role in the pathogenesis of heterotopic ossification by suppressing autophagy in ST2 receptor-expressing inflammatory cells.

Bacterial cellulose (BC) is a biomaterial with remarkable properties and versatile applications, synthesized by bacteria. Its high biocompatibility, biodegradability, and renewability render it an indispensable nanomaterial in the field of life sciences 23. Notably, the inherent 3D nanofibrous network of bacterial cellulose entraps substantial amounts of water (>99% water content), forming a bioactive functional hydrogel 24. Given its extensive application in wound healing, tissue regeneration, and drug delivery—owing to remarkable efficacy and exceptional drug-loading capacity 25-27—bacterial cellulose (BC) hydrogel represents a desirable platform to be leveraged for IL-33 scavenger loading and is expected to be effective in preventing heterotopic ossification.

In this study, we aimed to investigate the effects of the IL-33/ST2 axis on macrophage polarization and mast cell degranulation following soft tissue injury, as well as the underlying molecular mechanisms and therapeutic strategies. The novel mechanisms we identified could further substantiate the critical role of inflammation in the formation of trauma-induced heterotopic ossification and may provide new targets for the prevention and treatment of HO in the future.

## Materials and Methods

### Primary cell isolation and culture

We performed the isolation of bone marrow cells from 7-8-week-old C57BL/6 male mice based on methods described in the literature 30. Briefly, tibias and femurs were isolated from euthanized mice, soft tissues were removed, and a 26G needle was used to flush the bone marrow cavity. Next, the collected solution was transferred into a Centrifuge 5702 (Eppendorf AG, Germany) and centrifuged at 1000 revolutions per minute (rpm) for 5 minutes to remove impurities and obtain bone marrow cells. Thereafter, distinct methods were applied to induce bone marrow cell differentiation into Bone marrow-derived macrophages (BMDMs), Bone marrow-derived mast cells (BMMCs), as previously described 30-32.

#### Primary culture of BMDMs

Bone marrow cells were resuspended in complete RPMI 1640 medium supplemented with 10% fetal bovine serum (FBS) (Servicebio, China), 1% penicillin/streptomycin (Servicebio, China), and 40 ng/mL M-CSF (Peprotech, NJ) and plated in 10 cm cell culture plates. The culture medium was refreshed, and non-adherent cells were removed every 2-3 days during incubation. When cells reached 85% confluence, they were scraped and passaged, subsequently switching to a medium with a 20 ng/mL M-CSF concentration to maintain cell survival.

#### Primary culture of BMMCs

Bone marrow cells were cultured in complete RPMI 1640 medium supplemented with 10% FBS, 10 ng/mL recombinant murine IL-3 (ABclonal, China), and 20 ng/mL recombinant murine SCF (Novoprotein, China). The culture medium was refreshed, and non-adherent cells were carefully collected every 7 days during the purification process. The purity of BMMCs was assessed through toluidine blue staining and flow cytometry to analyze the expression of c-kit (CD117) and FcεRIα. Only cells with purity greater than 95% were used.

#### Primary culture of TDSCs

Tendon tissues were aseptically harvested from the Achilles tendons and patellar tendons of euthanized mice. After meticulous removal of the surrounding paratenon and fat, the tissues were minced into approximately 1 mm³ fragments and digested in a solution of 3 mg/mL collagenase type I (or type II/XI) at 37°C for 60-90 minutes. The digested tissue was then filtered through a 70 μm cell strainer. The resulting cell suspension was centrifuged, and the pellet was resuspended and cultured in complete DMEM/F-12 medium supplemented with 10% FBS and 1% penicillin/streptomycin. TDSCs within 3 passages were used in subsequent experiments.

The BMDMs and BMMCs used in our *in vitro* experiments were isolated from both wild-type (WT) and ST2 global knockout (ST2-/-) mice, while the TDSCs were isolated from WT mice only. All cells were cultured in a 37°C incubator with 5% CO₂.

#### BMDMs and BMMCs treatment

BMDMs and BMMCs were plated into 6-well plates and randomly divided into 6 groups: 1. Control group, IL-33 group (cells treated with recombinant murine IL-33 (rIL-33)), IL-33+ST2-/- group (cells from ST2-knockout mice treated with rIL-33); 2. IL-33 group (cells treated with rIL-33); 3. IL-33+LY group (cells treated with rIL-33 and LY294002 (PI3K inhibitor)), IL-33+AZD group (cells treated with rIL-33 and AZD5363 (AKT inhibitor)), IL-33+RAP group (cells treated with rIL-33 and Rapamycin (mTOR inhibitor)).

### The osteogenic differentiation of TDSCs

TDSCs were isolated using a modified enzymatic digestion protocol based on established methods. Briefly, tail tendons harvested from 2-3-week-old male C57BL/6 mice were minced into 1-2 mm³ fragments under sterile conditions. Tissue fragments underwent sequential digestion with 3 mg/mL collagenase type I (Worthington) and 4 mg/mL Dispase type II (Worthington) at 37°C for 2 h with gentle agitation. The resultant cell suspension was filtered through a 70 μm mesh, followed by centrifugation at 1000 rpm for 5 min to pellet cells. After two PBS washes, cells were resuspended in α-MEM complete medium supplemented with 10% FBS and maintained at 37°C in a 5% CO₂ humidified incubator, with medium refreshed every 48 hours. Cells below passage 3 were exclusively utilized for subsequent experiments. Each time the culture medium was refreshed, supernatant collected from BMDMs and BMMCs was supplemented.

ALP staining was performed using the BCIP/NBT Alkaline Phosphatase Color Development Kit (Beyotime, China) according to the manufacturer's instructions after 7 days of osteogenic induction. ALP activity was also quantified with the Alkaline Phosphatase Assay Kit (Nanjing Jiancheng Biotechnology Co Ltd, China). After 21 days of osteogenic differentiation, Alizarin Red S (ARS) staining was conducted with a 2% Alizarin Red S solution (pH 4.2) (Oricell, China). For ARS quantification, the ARS dye was dissolved using 10% cetylpyridinium chloride, and the resulting solution was transferred to a 96-well plate. Absorbance at 570 nm was measured with a SpectraMax i3x (Molecular Devices, Australia).

### Adenoviral transfection of cells

Adenovirus expressing the mCherry-GFP-LC3B fusion protein was synthesized by Beyotime (Shanghai, China). Adenoviral transfection was performed according to the manufacturer's instructions. Given the low infectivity of adenoviruses in inflammatory cells, we utilized an elevated MOI value (200 MOI) in conjunction with the viral transduction enhancer Polybrene (4μg/ml) in our experiments. The entire process lasted for 72 hours. Subsequently, a DMI8 microscope was used to detect the mean number of mCherry and GFP dots in cells.

### Cell immunofluorescence staining

After treatment, cells were fixed with 4% paraformaldehyde and subsequently permeabilized using 0.5% Triton X-100. Nonspecific binding sites were blocked with 1% bovine serum albumin (BSA), followed by corresponding primary antibodies at 4°C. Fluorescently conjugated secondary antibodies were then applied to label the cells under light-protected conditions. Nuclei were counterstained with 4,6-diamidino-2-phenylindole (DAPI), and fluorescence imaging was performed using an Olympus DP70 inverted microscope (Japan). The degranulation process of BMMCs was visualized using a laser confocal microscope (Nikon, Tokyo, Japan). Image J was used to calculate the mean fluorescence intensity of collagen I and TNC. The primary antibodies used in this study are listed in Supplementary Table S1.

### Flow cytometry analysis

Flow cytometry was utilized to assess alterations in surface markers of BMDMs. BMDMs were seeded into 6-well plates and treated with specific compounds. Cells were subsequently harvested using a cell scraper and washed three times with PBS. The collected cells were blocked with CD16/32 antibodies (Clone 93, Biolegend) on ice for 15 minutes to prevent nonspecific binding, followed by staining with BV421-labeled F4/80 (Clone BM8, Biolegend), PE-Dazzle594-labeled CCR7 (Clone 4B12, Biolegend), and PE-labeled MMR (Clone C068C2, Biolegend) antibodies under light-protected conditions for 30 minutes. After washing with PBS, surface marker expression was analyzed using a FACS AriaIII instrument (BD, New Jersey, USA), and data were analyzed with FlowJo software (Tree Star Inc., San Carlos, USA).

### Real-time quantitative polymerase chain reaction

Total RNA was extracted using TRIzol reagent (Invitrogen, USA) as per the manufacturer's protocol. Complementary DNA was synthesized through reverse transcription with M-MLV reverse transcriptase (Takara, Japan). Target gene mRNAs were quantified using SYBR Green Premix Ex Taq (Takara, Japan). Primers for the genes are detailed in Supplementary Table 3, with GAPDH serving as the housekeeping gene.

### Western blot (WB) analysis

Western blot analysis was executed following a previously established protocol 28. In brief, whole cells or tissue samples were disrupted on ice with RIPA lysis buffer (Epizyme, Shanghai) containing a protease inhibitor cocktail (Epizyme, Shanghai). Proteins from the supernatant were isolated via centrifugation, and their concentrations were assessed using a BCA assay. Uniform protein quantities (30 µg) were separated by electrophoresis on sodium dodecyl sulfate-polyacrylamide gels and subsequently transferred to polyvinylidene fluoride membranes. The membranes were then blocked with 5% nonfat milk or bovine serum albumin (BSA) for 1 hour and exposed to primary antibodies overnight at 4 °C. Following a 1-hour incubation with HRP-labeled secondary antibodies at room temperature, signals were visualized with an enhanced chemiluminescence reagent (Epizyme, Shanghai) and recorded using a ChemiDoc CRS imaging system (Bio-Rad, USA).

### Enzyme-linked immunosorbent assay (ELISA)

The secretion levels of cytokines TNF-α, IL-6, CCL2, TGF-β, and IL-10, along with the extent of β-hexosaminidase release, were quantified using ELISA kits (Anogen) according to the manufacturer's instructions.

### Transmission electron microscopy (TEM)

For autophagy evaluation, BMMCs, BMDMs and tissue samples were preserved in 2.5% glutaraldehyde buffered with 0.1 M sodium cacodylate at 4°C for 2 hours, then washed with 0.1 M sodium cacodylate supplemented with 7.5% sucrose (Sigma). Subsequently, the cells underwent post-fixation in 1% OsO4 solution (Sigma) for 1 hour and were dehydrated through an ethanol series. The specimens were encapsulated, stained with lead citrate, and analyzed via transmission electron microscopy.

### Animals and models

#### Animals

Wild-type (WT) C57BL/6 mice were acquired from the Shanghai laboratory animal center (SLAC), while the ST2-knockout (ST2-/-) mice (Strain NO. T005974) were purchased from GemPharmatech (Nanjing, China). Primer sequence used for genotyping ST2 knockout mice is described in Supplement Table S2. All animal experimental protocols were approved by the Institutional Animal Care and Use Committee (IACUC) of the Feinuo Phenotek Biotechnology (Shanghai) Co., Ltd, and experimental procedures were conducted in strict accordance with the guidelines of the National Institutes of Health Guide for the Care and Use of Laboratory Animals. All reports on animal studies complied with the ARRIVE guidelines.

#### Burn/Tenotomy Murine Model Establishment

7-8-week-old male C57BL/6 J mice (both WT type and ST2-knockout type) were selected for constructing the burn/tenotomy (B/T) model 31. Initially, following anesthesia with 1% pentobarbital sodium, a 1-cm longitudinal incision was created along the medial distal hindlimb surface of each mouse. Next, after blunt dissection of the tendon, a surgical blade was employed to sever the tendon at its midpoint. Subsequently, the skin was closed using 5-0 Vicryl sutures, ensuring the severed tendon remained encased within the skin to facilitate self-repair. Following tenotomy, an approximately 30% skin surface burn was induced using a 35g aluminum block, which was uniformly preheated to 60°C in a water bath and then applied to the shaved dorsal skin for 20 seconds. Sham surgery involved merely exposing the Achilles tendon and suturing the incision without inducing a burn injury.

#### Grouping

1.Sham group (sham surgery and local Achilles tendon injection of drug vehicle), Control group (burn/tenotomy and local Achilles tendon injection of drug vehicle), IL-33 group (burn/tenotomy and local Achilles tendon injection of rIL-33 (4 μg/ml every two days)), ST2-/- group (burn/tenotomy and local Achilles tendon injection of PBS using ST2-knockout mice).

2.IL-33+LY group (burn/tenotomy and local Achilles tendon injection of rIL-33 (4 μg/ml every two days) and PI3K inhibitors LY294002 (1.2 mg/kg)), IL-33+AZD group (burn/tenotomy and local Achilles tendon injection of rIL-33 (4 μg/ml every two days) and AKT inhibitors AZD5363 (1.5 mg/kg)), IL-33+Rap group (burn/tenotomy and local Achilles tendon injection of rIL-33 (4 μg/ml every two days) and mTOR inhibitors Rapamycin (2 mg/kg)). IL-33 is dissolved using PBS (Servicebio, China), while the three inhibitors were dissolved using DMSO (Solarbio, China). Local Achilles tendon injections of rIL-33 were administered every two days, while the inhibitors were injected daily into the same site. Both regimens continued until 3 weeks post-surgery. Except for the specifically noted ST2-/- group, all other groups utilized wild-type (WT) mice.

3.Control group (burn/tenotomy), BC group (burn/tenotomy and bacterial cellulose (BC) hydrogel scaffold implantation at the tendon rupture interface), BC+sST2 group (burn/tenotomy and bacterial cellulose (BC) hydrogel + sST2 composite scaffold implantation at the tendon rupture interface).

### The collection of human samples

This investigation was conducted with the approval of the Institutional Review Board of Shanghai Sixth People's Hospital, affiliated with Shanghai Jiao Tong University School of Medicine. Written informed consent was obtained from all participating patients or their immediate family members. The study enrolled healthy individuals aged 20 to 55 who had developed heterotopic ossification (HO) following prior internal fixation surgery for elbow fractures. None of the included patients had undergone local radiotherapy. HO specimens were collected during subsequent elbow arthrolysis procedures. Due to ethical considerations stipulating that surgical intervention be performed only upon HO maturation, all acquired samples represented the mature stage of the condition. Given the established soft tissue origin of HO—particularly from tendons—normal hamstring tendon tissues obtained from age-matched individuals undergoing anterior cruciate ligament reconstruction were utilized as control samples. Excess tendon tissue remaining after ligament reconstruction was harvested for this purpose.

### BC+ST2 composite scaffolds

#### Materials

Sodium hydroxide (NaOH; 99.0%), Glutaraldehyde (C5H8O2,25%) were purchased from Sinopharm Chemical Reagent Co., Ltd. ST2 was obtained from Novoprotein, China. Bacterial cellulose (BC) was obtained from Guilin Qihong Technology Co., LTD. All of the reagents were used as received without further purification. All water used was ultrapure (18.2 MΩ/cm), obtained from a Heal Force SMART Ultra-pure water system.

#### Preparation of BC hydrogel

The preparation of BC hydrogel was carried out by previously reported methods of synthesize BC hydrogel materials 27, 32. In a typical procedure, BC raw materials were boiled with 2% sodium hydroxide solution for 24 hours, then fully washed to neutral, spray drying to obtain BC powder. Then the BC powder was dissolved in ultra-pure water, fully stirred, and ultrasonically dispersed to obtain BC hydrogel with a solid content of 0.75 wt%.

#### Preparation of BC+ST2 composite scaffolds

The preparation of BC+ST2 composite scaffolds was carried out by previously reported methods of synthesize Arg modified collagen film materials 33. In a typical procedure, add ST2 to BC hydrogel, 10 ug/mL, stir thoroughly, and then incubated at 4℃ for 24 hours. It is then injected into a mold and freeze-dried to obtain a BC+ST2 scaffold.

#### Scanning electron microscope (SEM)

The macroscopic features of the samples were observed by SEM (ZEISS Sigma 560 VP) with an accelerating voltage of 1.0 kV and working distance at 5.6 mm to minimize charge accumulation. The scaffold sample is directly glued to double-sided carbon conductive adhesive and attach to the sample table. Then the sample table was placed in the scanning electron microscope chamber, vacuumed, opened the electron gun, opened the high pressure, and conducted the scanning test under the set conditions.

#### Fourier transform infrared spectrometer (FTIR)

FTIR patterns were recorded on a Thermelfeld iD1, Near-infrared spectrometer for solid measurement, operating temperature 5℃-40℃; The working relative humidity of the instrument is 5%-95%; Power consumption is less than 100VA. The wavenumber ranges from 450 to 4000 per cm.

#### ST2 release curve

The ST2 release curve was quantitatively characterized by ultraviolet spectrophotometer. First, the optimal excitation wavelength of ST2 was obtained by full-wave scanning, and then the normal saline immersed in BC+ST2 scaffold was scanned by the absorption curve for a long time using this wavelength to obtain the release curve of ST2 with increasing time. The absorption values in the figure correspond to the release concentration of ST2.

### Histological, immunohistochemical, and immunofluorescent staining

Tissues designated for evaluation were obtained at 7 days,3 weeks and 10 weeks following injury. Soft tissues extending from the tendomuscular junction to the enthesis were excised and fixed in 10% formalin. Samples retrieved at 10 weeks were subjected to a 14-day decalcification process using 10% EDTA. After embedding in paraffin, coronal sections of 5 μm thickness were crafted and affixed to glass slides. Staining with Hematoxylin and Eosin (HE), Safranin O/Fast Green (SO/FG) and Toluidine Blue (TB) proceeded according to the manufacturer's standard protocol. In the immunohistochemical staining procedure, sections underwent deparaffinization and rehydration via an alcohol gradient series. Antigen exposure was achieved in sodium citrate solution, after which sections were treated with 3% hydrogen peroxide for 15 minutes to suppress endogenous peroxidase activity. Blocking occurred with 10% serum for 1 hour, followed by overnight exposure to specific antibodies at 4°C. Sections were rinsed three times with PBST and then interacted with biotinylated secondary antibodies for 1 hour at room temperature. Signal revelation was accomplished by applying SAB complex and diaminobenzidine. Examination of all sections was conducted using an Olympus microscope. The immunofluorescent staining process replicated the rehydration, antigen retrieval, and blocking steps of immunohistochemistry. Sections were kept with specific antibodies overnight at 4 °C and subsequently processed with Alexa Fluor 488- or Cy3-conjugated secondary antibodies for 1 hour at 37 °C. Nuclear staining was performed with DAPI. Visualization was enhanced through pseudo-color techniques. Triple immunofluorescent staining employed tyramide signal amplification (TSA). Concisely, sections were deparaffinized and rehydrated prior to antigen retrieval and blocking. Primary antibodies were introduced sequentially and paired with horseradish peroxidase (HRP)-conjugated polymer. Each antibody's signal was rendered visible using CY3-TSA, FITC-TSA, or CY5-TSA. Antibody removal was executed between detection phases. Ultimately, nuclei were stained with DAPI, and the resulting slides were scanned with a Pannoramic digital scanner (Pannoramic P250; 3DHISTECH) or Tissue FAXS Spectra Platform. Primary antibodies utilized in this study are detailed in Table S1 (Supporting Information).

### Micro-CT imaging

At 10 weeks following burn/tenotomy, mice were euthanized, and hindlimbs from each group were harvested and fixed in 10% (v/v) formalin. After fixation for 48 hours, samples were analyzed using a high-resolution Micro-CT scanner, Skyscan 1176 (software version 1.1, build 6; Bruker, Kontich, Belgium). Imaging parameters were configured with an isometric resolution of 18 mm and a voltage of 70 kV. Three-dimensional visuals were generated and extracted via CTvox software (version unspecified). Quantification of bone volume was conducted with CTan software (version 1.15.4.0+; Bruker) following a previously outlined method, 14 wherein high-density masses within soft tissue exceeding a Hounsfield unit of 272 were identified as aberrant bone.

### Statistical analysis

Data processing was conducted using GraphPad Prism 10. Results are presented as means ± standard deviations (SD). Normality was evaluated with the Shapiro-Wilk test, while variance homogeneity was assessed via one-way analysis of variance (ANOVA). Group comparisons were executed through one-way, with Tukey's test applied for subsequent pairwise analysis when data followed a normal distribution. For non-normally distributed data, the Kruskal-Wallis test was utilized. Categorical data comparisons were performed using the chi-square test. A significance threshold of P<0.05 was established, and two-tailed testing was implemented. Sample sizes were predetermined using PASS software version 15 (NCSS Statistical Software, UT), based on preliminary findings, with α set at 0.05 and β at 0.1. Each experiment was independently replicated at least three times, incorporating both biological and technical repeats.

## Result

### IL-33/ST2 signaling axis is activated in the early phase of HO formation and drives macrophage M2 polarization and mast cell activation

We first performed preliminary experiments on surgically excised human heterotopic ossification (HO) specimens. In contrast to normal human tendon tissue, immunofluorescence analysis revealed a marked upregulation of IL-33 in ectopic bone samples, accompanied by a substantial overexpression of its receptor ST2 on CD206⁺ macrophages and tryptase⁺ mast cells. (Fig. S1 A-C) These observations led us to hypothesize that the IL-33/ST2 signaling axis may play a critical role in the pathogenesis of heterotopic ossification, although its precise mechanism remained to be elucidated.

**Figure 1:**
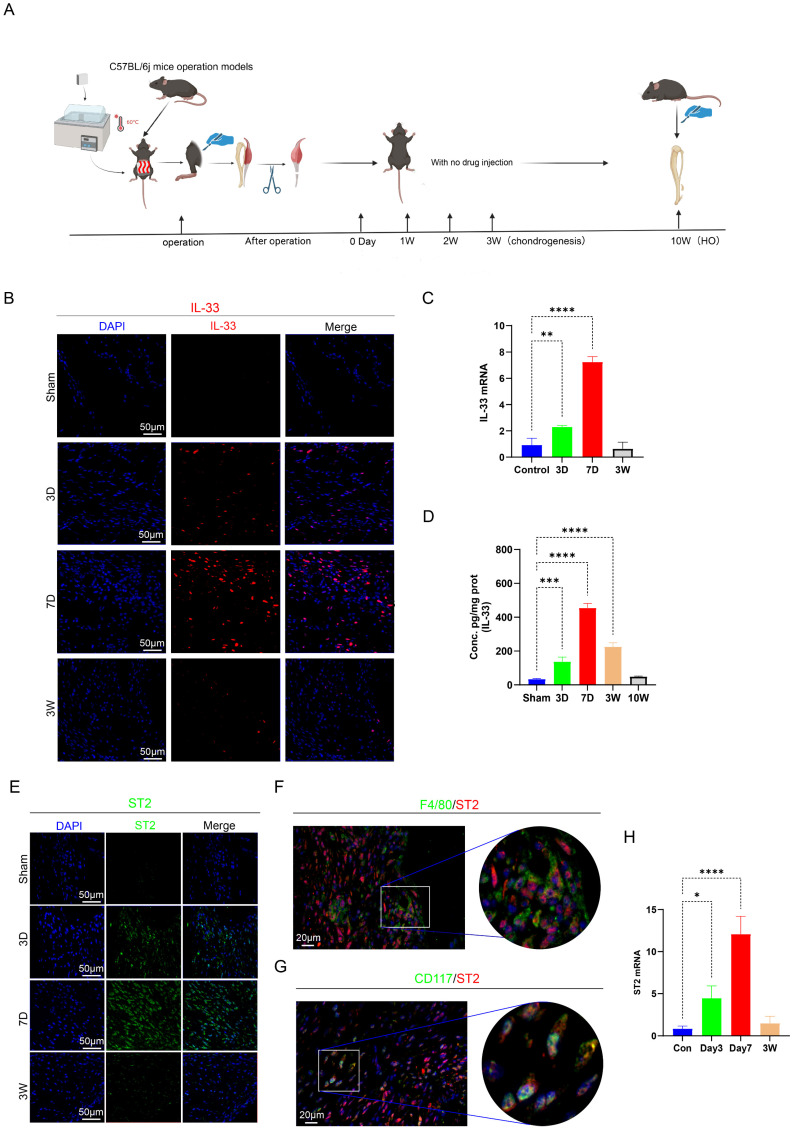
** IL-33/ST2 axis were actived after Burn/Tenotomy. (A)** Establishment process of the burn/tenotomy model. **(B, E)** Immunofluorescence staining of IL-33 and ST2 at indicated time points. **(C, H)** Relative gene expression of IL-33 and ST2 evaluated by qRT-PCR. **(D)** IL-33 protein expression levels assessed by ELISA. **(F, G)** Double fluorescence staining of ST2 and the macrophage marker F4/80 or the mast cell marker CD117 at 7 days post-trauma. N=3/group, *P < 0.05, **P < 0.01, ***P < 0.001, ****P < 0.0001. All data are presented as mean ± SD.

To further investigate the underlying molecular pathways, we established the burn/tenotomy model using C57BL/6 mice (wild-type (WT) and ST2 global knockout (ST2-/-) mice). The model construction process and the key stages of HO development were described (Fig. 1A). Given that IL-33, as an alarmin, is rapidly released post-injury, we assumed it triggered the early inflammatory response during HO formation. As expected, immunofluorescence staining, qRT-PCR, and ELISA results all revealed that IL-33 appeared early at 3 days, peaked at 7 days, and persisted for up to 3 weeks after the tendon injury (Fig. 1B-D). Correspondingly, the expression of IL-33's receptor ST2 follows a similar timeline. Furthermore, immunofluorescence staining experiments confirmed that on day 7, the ST2 receptor was significantly expressed on F4/80+ macrophages and CD117+ mast cells (Fig. 1E-H). The above results confirmed that macrophages and mast cells might be the effector cells that responded to the surged IL-33 expression. In addition, day 7 is an optimal time point for observation of inflammatory changes in the following experiments.

**Figure 2:**
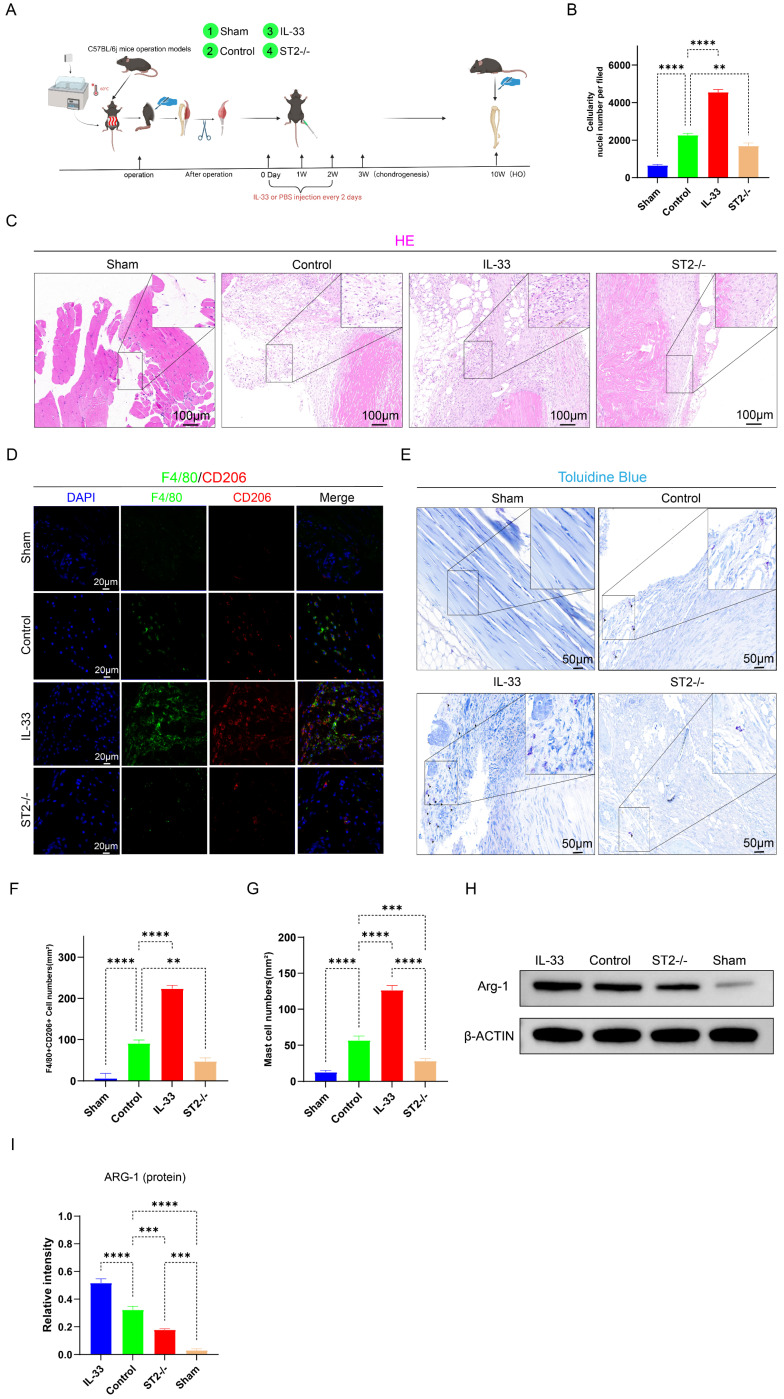
** IL-33/ST2 Axis Locally Induces M2 Macrophages Polarization and Mast Cells Activation after Soft Tissue Injury. (A)** In vitro experimental treatment design. **(B, C)** H&E staining showing injured tissue cellularity at 7 days post-burn/tenotomy. **(D)** Double fluorescence staining of F4/80 and CD206 at 7 days post-trauma. **(E, I)** Toluidine blue (TB) staining showing mast cell accumulation and activation at 7 days post-burn/tenotomy. **(F)** Quantitative analysis of double-positive M2 macrophages revealed by fluorescence staining. **(G, H)** Arg-1 protein expression detected by western blot. N=3/group, *P < 0.05, **P < 0.01, ***P < 0.001, ****P < 0.0001. All data are presented as mean ± SD.

Next, we constructed burn/tenotomy models using WT and ST2-/- mice with or without IL-33 injection (Fig. 2A). At 7 days post-injury, H&E staining showed that after burn/tenotomy injury, compared to WT mice, ST2-/- mice exhibited a marked reduction in inflammatory cell numbers. In contrast, WT mice treated with recombinant IL-33 (rIL-33) exhibited a more severe inflammatory response at the injured tendon (Fig. 2B-C), as indicated by the more abundant inflammatory cell infiltration in H&E staining. Immunofluorescence staining for M2 macrophage markers F4/80 and CD206 was performed to examine whether alterations in macrophage polarization status correlated with IL-33 during HO formation. The results showed that IL-33 stimulated M2 macrophage polarization, and this was further confirmed by Western blot (WB) experiments (Fig. 2D, F, H-I). Additionally, mast cell recruitment and activation were enhanced in rIL-33-treated mice but diminished in ST2-/- mice compared with wild-type controls, which was revealed by toluidine blue (TB) staining (Fig. 2E, G).

Building on evidence from both human specimens and animal models, we collectively demonstrate that the IL-33/ST2 axis orchestrates the early inflammatory response in heterotopic ossification by promoting M2 macrophage polarization and mast cell activation.

### IL-33/ST2 axis suppresses autophagy in macrophages and mast cells via an ST2-dependent mechanism

**Figure 3:**
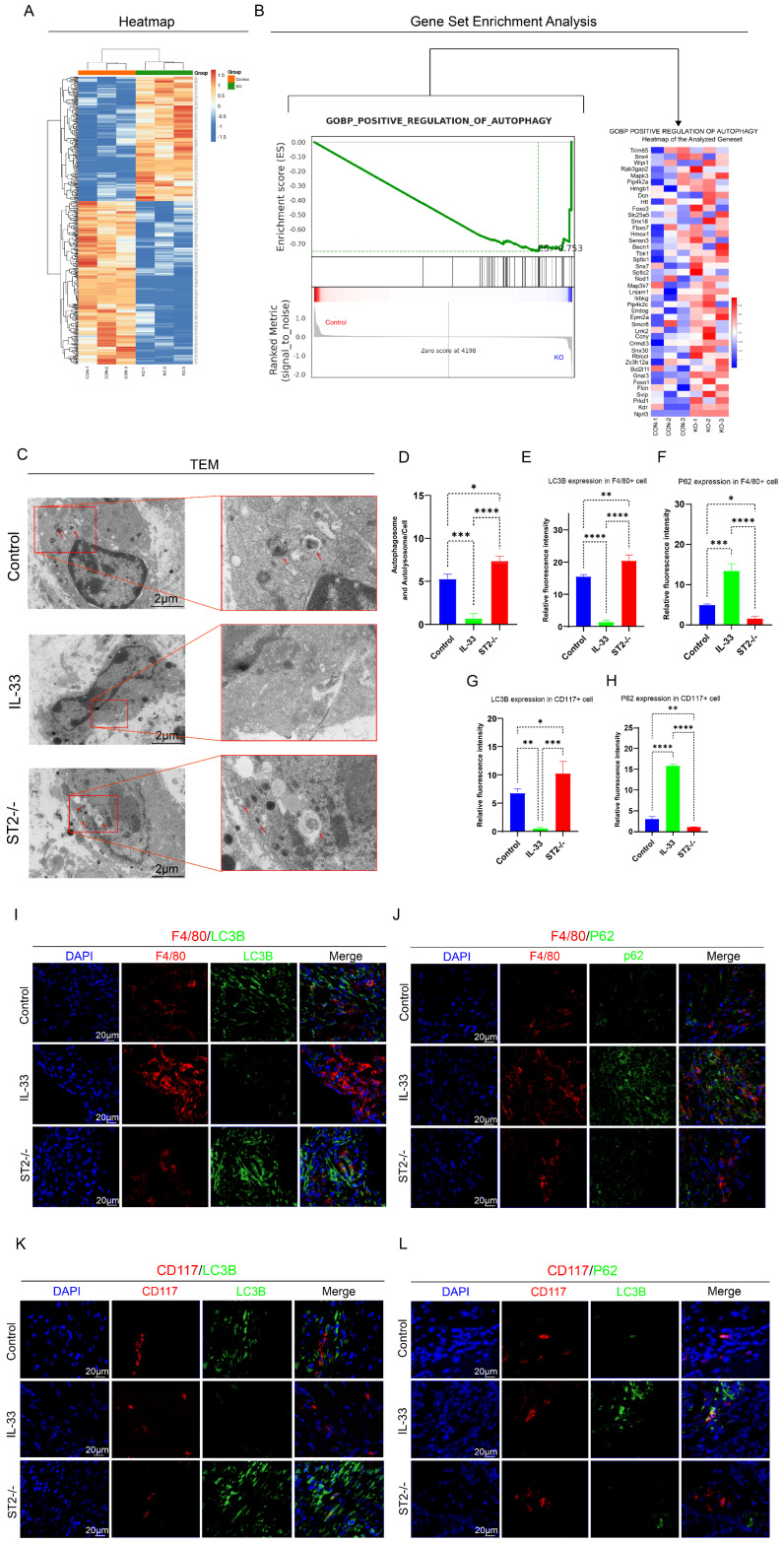
** IL-33/ST2 Inhibits Autophagy in Macrophages and Mast Cells After Trauma. (A)** Heatmap of differential expression revealed by proteomics between control and ST2-knockout groups. **(B)** GSEA analysis associated with positive regulation of autophagy between control and ST2-knockout groups.** (C, D)** Representative images and quantification of autophagosomes and autolysosomes in inflammatory cells around the injured tissue by TEM. **(E, F, I, J)** Double fluorescence staining of F4/80 and LC3B or P62 for macrophage autophagy around the injured tissue.** (G, H, K, L)** Double fluorescence staining of CD117 and LC3B or P62 for mast cell autophagy around the injured tissue. N=3/group, *P < 0.05, **P < 0.01, ***P < 0.001, ****P < 0.0001. All data are presented as mean ± SD.

Next, to explore the molecular mechanisms of IL-33-dependent alteration of inflammatory cell activity states, proteomics was performed on lesion tissues collected at 7 days post-injury—a time point corresponding to the peak of the inflammatory response during HO formation—from the Sham, Control (WT mice), and ST2-/- (ST2-knockout mice) groups. Subsequently, a differential analysis between the Control group and the ST2-/- group was further performed (Fig. 3A). Of particular importance to this study were the findings of upregulated gene expression related to autophagy in the knockout group, leading to a marked enrichment of genes involved in the positive regulation of autophagy in the GSEA analysis (Fig. 3B-C).

Firstly, transmission electron microscopy (TEM), the gold standard for autophagy detection, revealed a higher abundance of autophagosomes and autolysosomes in the ST2-/- group compared to the Control group, whereas no obvious autophagy was observed in the IL-33 group (Fig. 3C-D). Moreover, immunofluorescence staining for LC3B and P62 proteins was performed to assess autophagic activity in mast cells and macrophages within the injured tissue (Fig. 3E, F, I, J for macrophages; Fig. 3G, H, K, L for mast cells). LC3B expression in both macrophages and mast cells decreased sharply in the IL-33 group compared to controls, but increased in the ST2-/- group. In contrast, P62 expression increased in the IL-33 group but decreased in ST2-/- mice compared to controls. These results further validated the findings derived from proteomics analysis and TEM. Collectively, IL-33 suppresses autophagy in inflammatory cells expressing the ST2 receptor after trauma.

**Figure 4:**
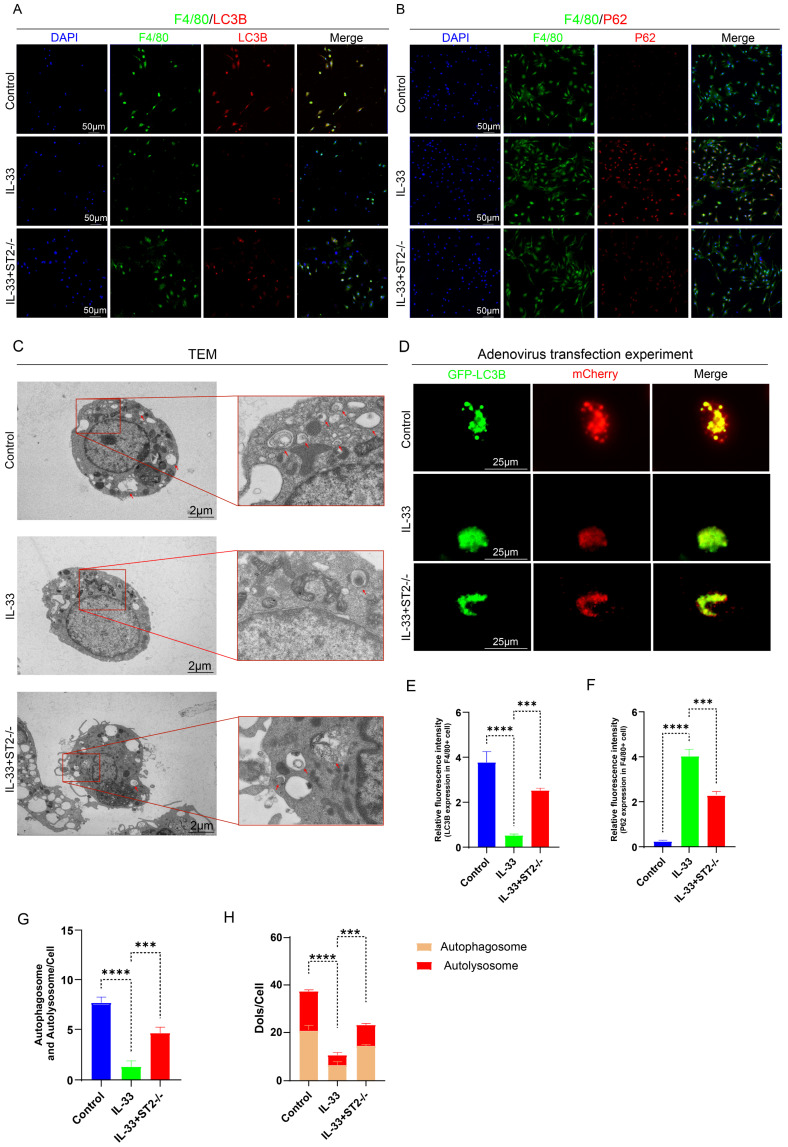
** IL-33/ST2 inhibits autophagy in BMDMs *in vitro*.** BMDMs from the IL-33 and IL-33+ST2-/- groups were treated with rIL-33 (50 ng/ml) for 8 hours. **(A, B, E, F)** Double fluorescence staining of F4/80 and LC3B or P62 for BMDMs. **(C, G)** Representative images and quantification of autophagosomes and autolysosomes in BMDMs by TEM. **(D, H)** BMDMs transfected with Ad-mCherry-GFP-LC3B and mean numbers of mCherry and GFP dots per cell. N=3/group, *P < 0.05, **P < 0.01, ***P < 0.001, ****P < 0.0001. All data are presented as mean ± SD.

To more directly and precisely investigate the impact of IL-33 on autophagy in macrophages and mast cells, we then isolated primary BMDMs and BMMCs for *in vitro* experiments. As mentioned before, we divided the BMDMs and BMMCs each into three groups: the Control group, the IL-33 group, and the IL-33+ST2-/- group. Immunofluorescence staining, TEM, and mCherry-GFP-LC3 reporter assay, were used respectively to detect autophagy levels in these two inflammatory cell types. After an 8-hour treatment with recombinant IL-33 (rIL-33) (50 ng/ml), LC3B expression in F4/80+ WT BMDMs (isolated from WT mice) was significantly decreased and P62 expression was markedly increased, indicating autophagy inhibition, as confirmed by immunofluorescence staining. However, these IL-33-induced autophagic changes were not prominent in F4/80+ ST2-/- BMDMs (isolated from ST2-/- mice) (Fig. 4A, B, E, F). Moreover, TEM showed that the number of autophagosomes and autolysosomes in WT BMDMs was sharply reduced, while ST2-/- BMDMs showed a mild decrease after IL-33 addition (Fig. 4C, G). Lastly, a tandem fluorescence Ad-mCherry-GFP-LC3B reporter system was chosen for distinguishing autophagic flux. Following infection of cells with the Ad-mCherry-GFP-LC3B adenovirus, under non-autophagic conditions, fluorescence microscopy revealed mCherry-GFP-LC3B distributed diffusely in the cytoplasm as yellow fluorescence. In contrast, under autophagic conditions, mCherry-GFP-LC3B aggregates on the autophagosomal membrane, appearing as yellow punctate structures. Upon fusion of autophagosomes with lysosomes, the GFP fluorescence is partially quenched due to the acidic environment, resulting in the appearance of red punctate structures. The results showed that IL-33 both markedly suppressed the GFP-LC3B puncta and autophagic flux in WT BMDMs, whereas the reduction in ST2-/- BMDMs was less substantial (Fig. 4D, H).

**Figure 5:**
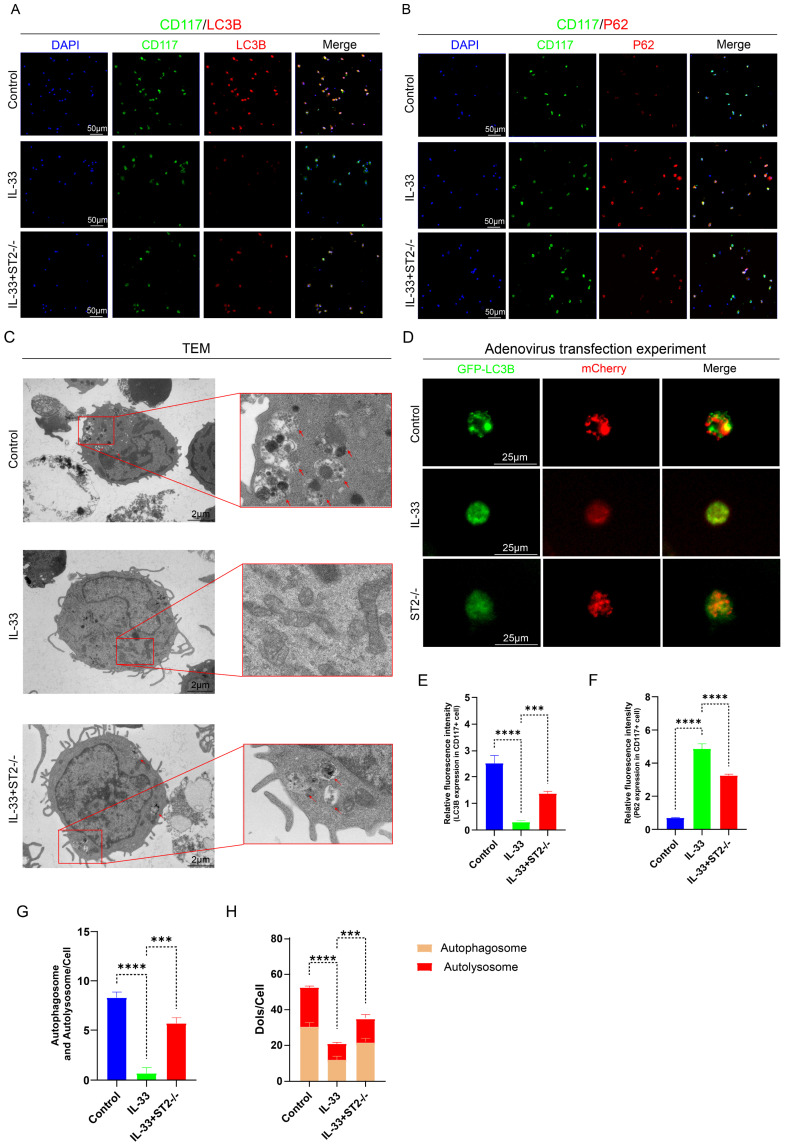
** IL-33/ST2 inhibits autophagy in BMMCs *in vitro*.** BMMCs from the IL-33 and IL-33+ST2-/- groups were treated with rIL-33 (50 ng/ml) for 8 hours. **(A, B, E, F)** Double fluorescence staining of CD117 and LC3B or P62 for BMMCs. **(C, G)** Representative images and quantification of autophagosomes and autolysosomes in BMMCs by TEM. **(D, H)** BMMCs transfected with Ad-mCherry-GFP-LC3B and mean numbers of mCherry and GFP dots per cell. N=3/group, *P < 0.05, **P < 0.01, ***P < 0.001, ****P < 0.0001. All data are presented as mean ± SD.

The same experiments were repeated in BMMCs and yielded results highly similar to those observed in BMDMs. IL-33 treatment significantly suppressed autophagy in WT BMMCs, yet exerted minimal effect on ST2-/- BMMCs (Fig. 5A-H).

Thus far, we have successfully validated the IL-33-mediated suppression of autophagy in inflammatory cells through the ST2 receptor.

### PI3K/AKT/mTOR pathway initiated by IL-33/ST2 axis was responsible for autophagy inhibition

The PI3K/AKT/mTOR signaling cascade serves as a critical regulator of cellular autophagy 34-35. Under physiological conditions, activation of PI3K triggers the phosphorylation of AKT, thereby activating mTOR, which is widely known for suppressing the initiation of autophagy. Accordingly, we hypothesize that IL-33 potentially mediates the activation of the PI3K/AKT/mTOR signaling pathway through the ST2 receptor. As before, we established three groups: the Control group, the IL-33 group, and the ST2-/- group (Fig. 2A). Western blotting analysis and immunofluorescence staining of injured tissues confirmed our hypothesis (Fig. S2A-I, supporting information), showing that the expression level of p-PI3K, p-AKT and p-mTOR in both macrophages and mast cells upregulated in the IL-33 group but downregulated in the ST2-/- group.

*In vitro* experiments were also performed, yielded similar results, as revealed by WB and immunofluorescence staining. The expression levels of phosphorylated markers in the control group cells were the lowest observed. Moreover, treatment with recombinant IL-33 (rIL-33) (50 ng/ml) for 8 hours sharply increased p-AKT and p-mTOR levels in both WT BMDMs and BMMCs; however, cells from ST2-/- group displayed less responsiveness to this stimulus (Fig. S3A-H for BMDMs; Fig. S3I-P for BMMCs).

Based on these *in vivo* and *in vitro* findings, we demonstrate that IL-33/ST2 signaling activates the PI3K/AKT/mTOR pathway, leading to autophagy suppression in both macrophages and mast cells.

### Pharmacological inhibition of PI3K/AKT/mTOR signaling reverses IL-33-induced autophagy suppression

**Figure 6:**
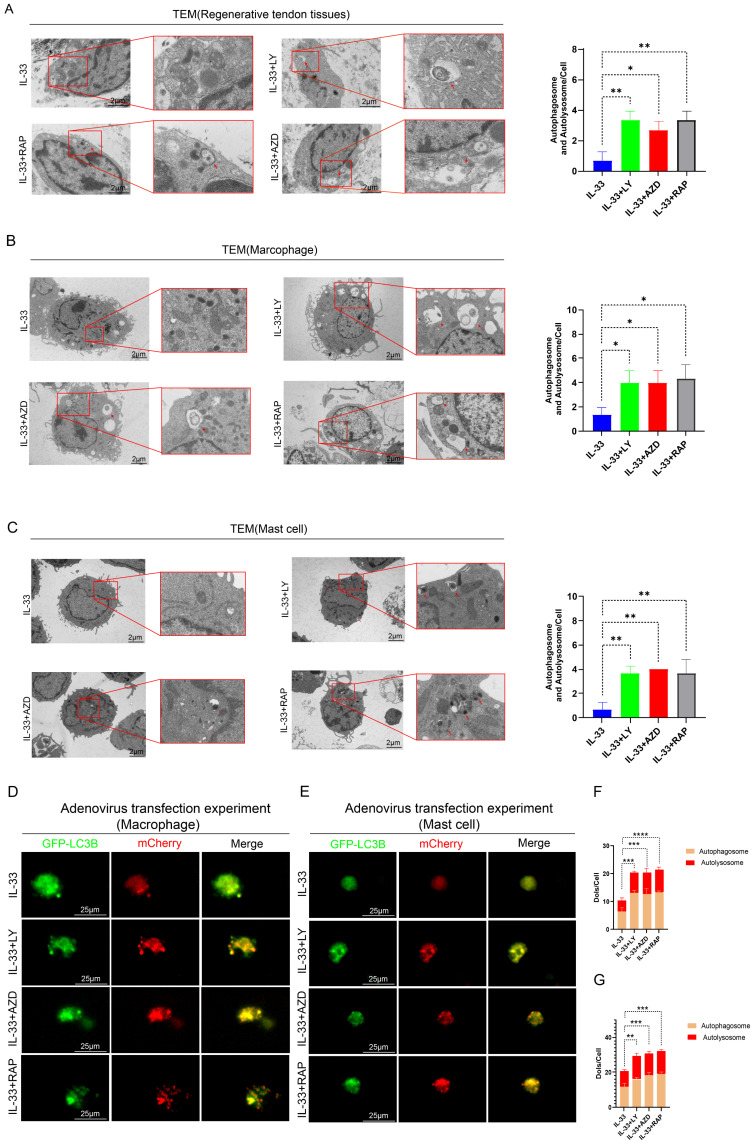
** Impaired autophagy can be restored by PI3K/AKT/mTOR pathway inhibitors both *in vivo* and *in vitro*.** In the *in vivo* experiment, in addition to IL-33 injection, each group received a single local injection of PI3K inhibitor (LY294002), AKT inhibitor (AZD5363), or mTOR inhibitor (Rapamycin) into the injured tendon respectively, while in the *in vitro* experiment, BMDMs and BMMCs from the IL-33+LY, IL-33+AZD, and IL-33+Rap groups were treated with the PI3K inhibitor, AKT inhibitor, or mTOR inhibitor for 8 hours, respectively. **(A)** Representative images and quantification of autophagosomes and autolysosomes in inflammatory cells around the injured tissue at 7 days post-trauma by TEM. **(B)** TEM results from BMDMs *in vitro*. **(C)** TEM results from BMMCs *in vitro*. **(D, F)** BMDMs transfected with Ad-mCherry-GFP-LC3B and the mean number of mCherry and GFP dots per cell. **(E, G)** Results from BMMCs transfected with Ad-mCherry-GFP-LC3B. N=3/group, *P < 0.05, **P < 0.01, ***P < 0.001, ****P < 0.0001. All data are presented as mean ± SD.

To further validate the critical role of the PI3K/AKT/mTOR pathway in autophagy suppression during HO formation, we designed rescue experiments. Three experimental groups were established; in addition to IL-33 injection, each group received a local injection of a PI3K inhibitor (LY294002), an AKT inhibitor (AZD5363), or an mTOR inhibitor (Rapamycin) into the injured tendon respectively (not shown in figures). Transmission electron microscopy (TEM) analysis of the tissue revealed that IL-33-induced autophagy suppression can be partially reversed by the three inhibitors targeting this pathway (LY294002, AZD5363, and Rapamycin), with no statistically significant differences observed among the outcomes of the three inhibitor groups (Fig. 6A). The expression levels of LC3B and P62 in CD117+ cells and F4/80+ cells, as revealed by immunofluorescence staining, were shown in the appendix (Fig. S4A-H). Inhibitor treatment concomitantly reversed the decreased LC3B and increased P62 levels caused by rIL-33 in both cell types.

*In vitro* rescue experiments on autophagy demonstrated consistent results. BMDMs and BMMCs were randomly allocated into three restoration groups, each receiving a specific inhibitor in combination with IL-33 (50 ng/ml) for 8 hours. TEM analysis indicated an increased presence of vacuoles in restoration groups compared to the IL-33-only control group (Fig. 6B for macrophages; Fig. 6C for mast cells). Additionally, adenovirus-mediated transfection experiments employing the Ad-mCherry-GFP-LC3B reporter system revealed that inhibitors of PI3K, AKT or mTOR not only restored autophagy initiation but also augmented autophagic flux irrespective of in macrophages or mast cells (Fig. 6D, F for macrophages; Fig. 6E, G for mast cells).

Together, these rescue experiments demonstrate that targeted inhibition of the PI3K/AKT/mTOR pathway effectively restores autophagic activity in both macrophages and mast cells, thereby establishing this cascade as the principal mechanistic link between IL-33/ST2 signaling and autophagy suppression during heterotopic ossification.

### Suppression of IL-33-mediated autophagy by PI3K/AKT/mTOR signaling inhibition reversed M2 macrophage polarization during trauma-induced heterotopic ossification

**Figure 7:**
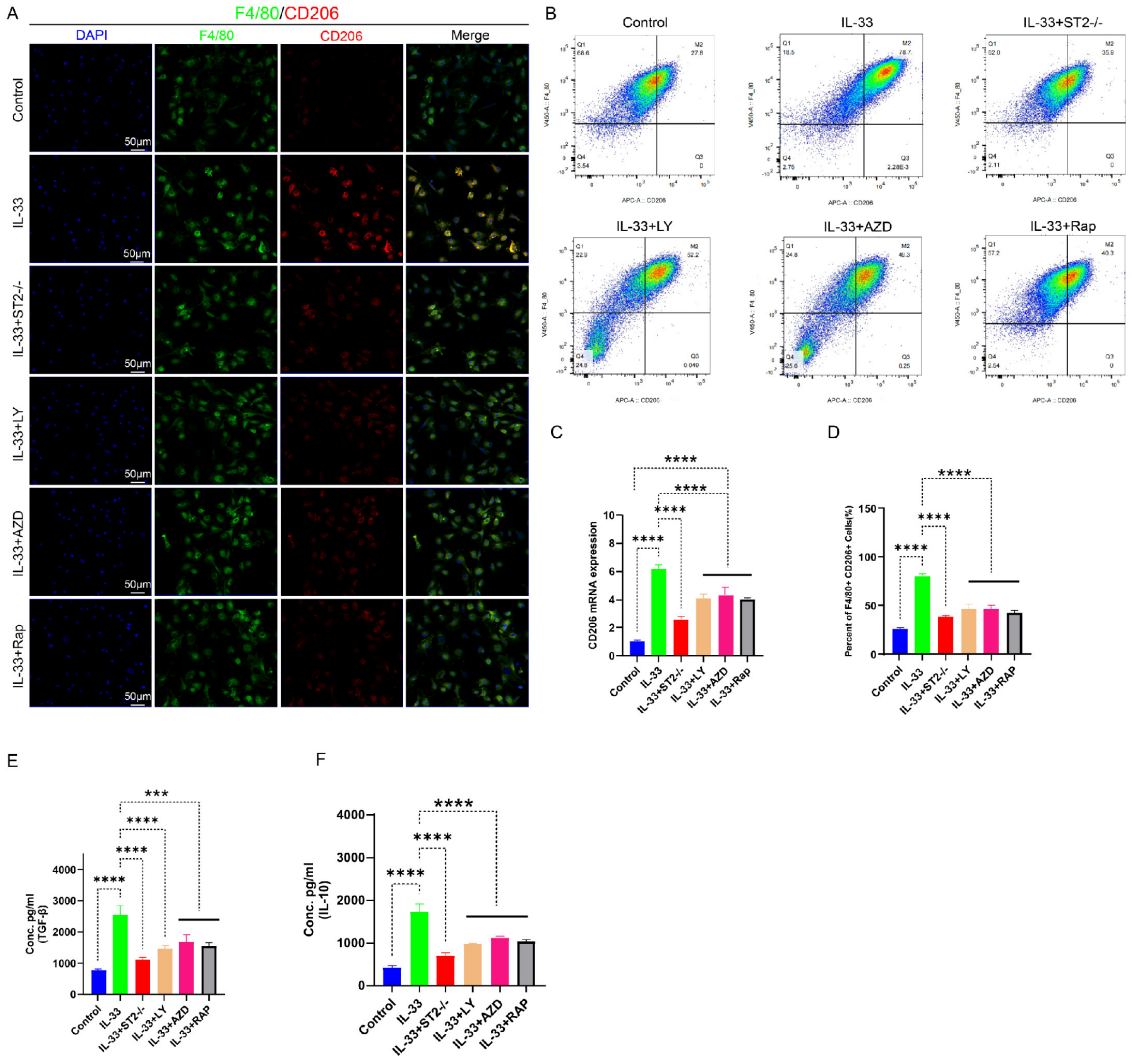
** IL-33-mediated autophagy suppression drove M2 macrophage polarization, which could be reversed by PI3K/AKT/mTOR pathway inhibitors. BMDMs were treated as described previously. (A)** Immunofluorescence staining of F4/80 and CD206. **(B)** Flow cytometry analysis of F4/80 and CD206. **(C)** CD206 mRNA expression detected by qRT-PCR. **(D)** Bar graphs illustrating the percentage of F4/80 and CD206 double-positive cells. **(E, F)** Inflammatory cytokines in the supernatants of BMDMs upon different treatments were examined by ELISA. N=3/group, *P < 0.05, **P < 0.01, ***P < 0.001, ****P < 0.0001. All data are presented as mean ± SD.

For *in vitro* experiments, we divided the primary BMDMs into six experimental groups: the Control group, the IL-33 group (treated with rIL-33), the IL-33+ST2-/- group (ST2-knockout BMDMs treated with rIL-33), the IL-33+LY group (treated with LY294002 and rIL-33), the IL-33+AZD group (treated with AZD5363 and rIL-33), and the IL-33+Rap group (treated with Rapamycin and rIL-33). Subsequently, we evaluated the polarization status of macrophages 24 hours post-drug treatment. The qRT-PCR results were consistent with those of *in vivo* experiments: When compared to the control group, the IL-33 group exhibited a significant increase in CD206 mRNA expression; however, ST2 receptor-deficient macrophages displayed diminished responsiveness to rIL-33 stimulation, reflected by a significant reduction in CD206 mRNA expression when compared to the IL-33 group. Furthermore, PI3K, AKT, and mTOR inhibitors significantly reversed these IL-33-induced changes in transcriptional level of CD206 (Fig. 7C). Immunofluorescence staining results for CD206 exhibited high consistency with qRT-PCR analysis outcomes (Fig. 7A). To more accurately acquire the polarization status of macrophage, we utilized flow cytometry to analyze the proportion of F4/80+CD206+ cells in the six groups. Recombinant IL-33 (rIL-33) significantly elevated the proportion of M2-polarized macrophages in comparison to the control group, whereas the proportion of M2 macrophages in the IL-33-treated ST2-knockout (IL-33+ST2-/-) group was marginally higher than that in the untreated control group. Similarly, the three pathway-specific inhibitors significantly reversed IL-33-induced changes to differing extents (Fig. 7B, D). Finally, we utilized ELISA to quantify the protein expression levels of TGF-β and IL-10 secreted by M2 macrophages in each group. The results were consistent with those reflected by immunofluorescence staining, qRT-PCR, and flow cytometry, which showed M2 macrophage-related cytokines were enhanced after IL-33 stimulation but downregulated upon ST2-knockout or three pathway inhibitors addition (Fig. 7E-F).

As our prior *in vivo* studies have established that IL-33 promoted macrophage polarization toward the M2 phenotype by suppressing autophagy post-trauma, we next conducted supplementary autophagy rescue experiments using PI3K, AKT, and mTOR inhibitors to further elucidate this mechanism. Immunofluorescence staining results revealed a marked reduction in F4/80+CD206+ cells surrounding the damaged tendon in groups treated with PI3K, AKT, and mTOR inhibitors, respectively, compared to the IL-33 control group (Fig. S5A, C). qRT-PCR analysis demonstrated consistent CD206 mRNA expression change patterns with the immunofluorescence staining results (Fig. S4D).

Based on these findings, we demonstrate that IL-33 drives macrophage M2 polarization through ST2-dependent activation of the PI3K/AKT/mTOR pathway, and this pro-polarizing effect can be effectively reversed by specific pathway inhibitors.

### IL-33/ST2 axis promotes mast cell degranulation through autophagy suppression

Upon stimulation by allergens, cytokines, or physical injury, mast cells release the contents of their intracellular granules, encompassing histamine, proteases, cytokines (e.g., IL-6, TNF-α), and chemokines 13,36. Post-trauma, these bioactive mediators liberated via mast cell degranulation rapidly elicit a localized inflammatory response, thereby playing a pivotal role in key pathophysiological processes, including cytokine storms, allergic reactions, and tissue repair following injury 37-39. Prior toluidine blue (TB) staining of damaged tissue lacked sufficient clarity and precision in evaluating mast cell degranulation responses; thus, we concentrated on elucidating the effects of IL-33/ST2-mediated autophagy suppression on mast cell degranulation via *in vitro* experiments using BMMCs. We employed the same experimental grouping design as applied to BMDMs.

**Figure 8:**
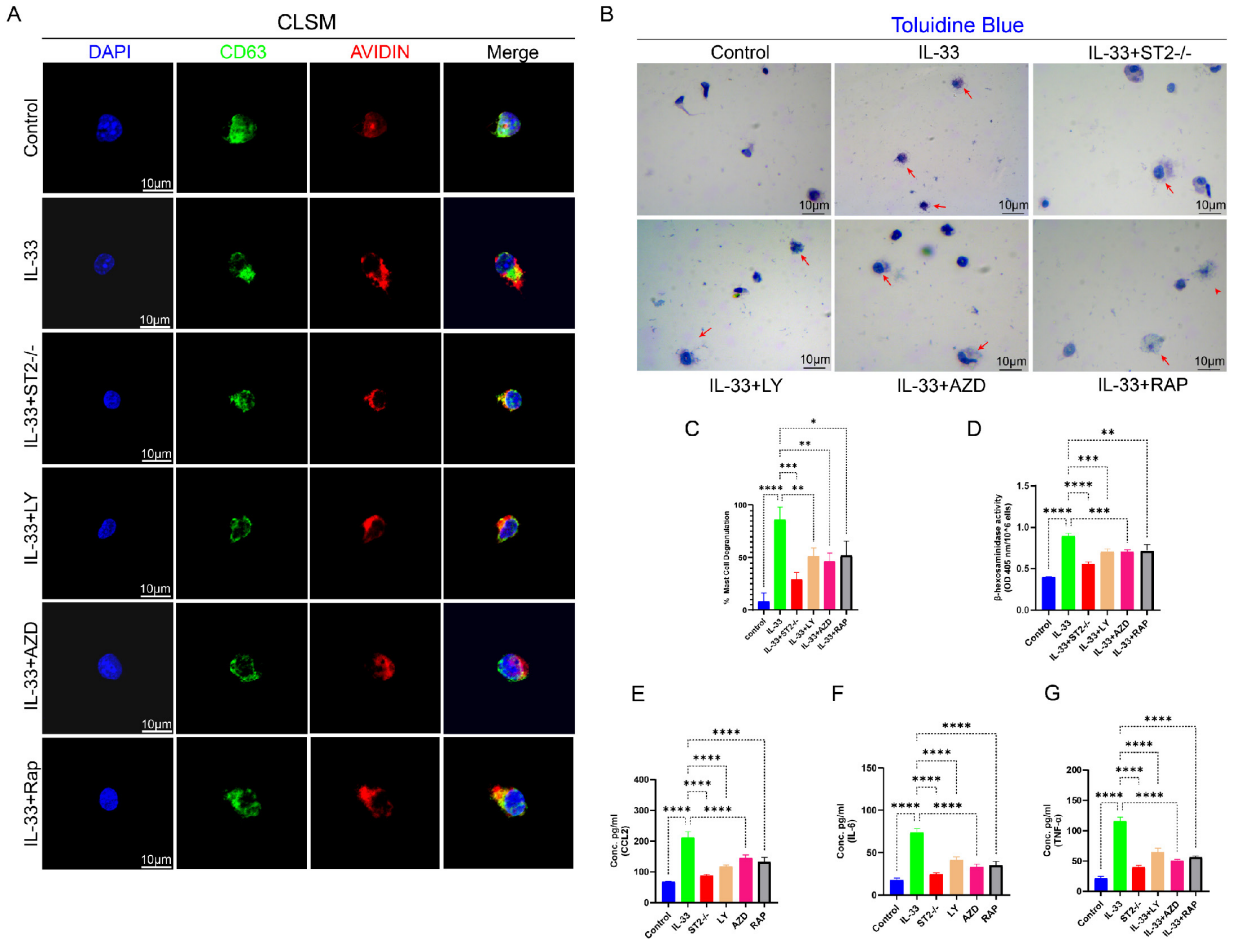
** IL-33-mediated autophagy suppression drove mast cell degranulation, which could be reversed by PI3K/AKT/mTOR pathway inhibitors. BMMCs were treated as described previously. (A)** Images from BMMCs stained with anti-CD63 antibody and avidin-fluorescein isothiocyanate under CLSM. **(B)** Toluidine blue staining for observation of BMMC degranulation. **(C)** Bar graphs illustrating the percentage of degranulated BMMCs. **(D)** Amount of released β-hexosaminidase after different treatments. **(E, F, G)** Inflammatory cytokines in the supernatants of BMMCs upon different treatments were examined by ELISA. N=3/group, *P < 0.05, **P < 0.01, ***P < 0.001, ****P < 0.0001. All data are presented as mean ± SD.

CD63, a tetraspanin protein, is widely expressed on the membranes of intracellular vesicles (e.g., lysosomes, exosomes, and secretory granules 39. In mast cells, CD63 exhibits a strong association with the secretory granules released during degranulation, rendering it a widely utilized surface marker for assessing mast cell degranulation, particularly in flow cytometry and immunofluorescence assays, where it facilitates the detection of dynamic changes in vesicular membrane protein expression post-degranulation 36. The intracellular level of avidin (a heparin-specific probe) in mast cells is routinely employed as an indicator to evaluate the degree of degranulation 41. BMMCs from each experimental group were stained with anti-CD63 antibody, a specific probe for mast cell granule membranes, and avidin-fluorescein isothiocyanate (FITC) to assess degranulation status. A decrease in avidin expression levels within CD63+ vesicles signify that degranulation has taken place following stimulation. Subsequently, we employed Confocal Laser Scanning Microscopy (CLSM) to perform a more direct microscopic examination of stained mast cell. In the Control group, the red fluorescence associated with avidin expression exhibited substantial colocalization with the green fluorescence of CD63 expression, suggesting that the cell had not undergone degranulation, while in the IL-33 group, a significant release of avidin from the vesicles was detected under CLSM. However, ST2-knockout BMMCs displayed reduced responsiveness to IL-33 stimulation (Fig. 8A). Toluidine blue staining demonstrated highly consistent results. Upon microscopic examination, the IL-33 group exhibited the greatest proportion of degranulated cells, followed by the IL-33+ST2-/- group, whereas the control group showed the lowest proportion of degranulated cells (Fig. 8B, C). The extent of mast cells degranulation was also assessed by quantifying the release of β-hexosaminidase in each group (Fig. 8D). Inflammatory mediators released during mast cell degranulation, such as CCL2, IL-6, and TNF-α, were also quantified using ELISA (Fig. 8E-G). The results demonstrated consistency with observations from CLSM and toluidine blue staining, which revealed that IL-33 treatment induced mast cell degranulation via the ST2 receptor.

Next, we performed *in vivo* and *in vitro* autophagy rescue experiments to further investigate the role of PI3K/Akt/mTOR-mediated autophagy in mast cell degranulation. In *in vivo* experiments, PI3K, AKT, and mTOR inhibitors markedly attenuated the IL-33-induced recruitment and activation of mast cells around the injured tissue (Fig. S4B, E). In *in vitro* experiments, inhibitors suppressed the stimulatory effects of IL-33 on BMMCs, as demonstrated by a reduction in degranulation. These findings were substantiated by confocal laser scanning microscopy (CLSM), toluidine blue staining, ELISA, and the β-hexosaminidase release quantification assay (Fig. 8A-G).

Collectively, these findings established that IL-33/ST2 signaling drives mast cell degranulation via autophagy inhibition, and pharmacological targeting of the downstream PI3K/AKT/mTOR pathway effectively attenuates this process both *in vitro* and *in vivo*.

### Inhibition of autophagy by PI3K/AKT/mTOR signaling attenuated inflammatory induced osteogenic healing during trauma-induced heterotopic ossification

**Figure 9:**
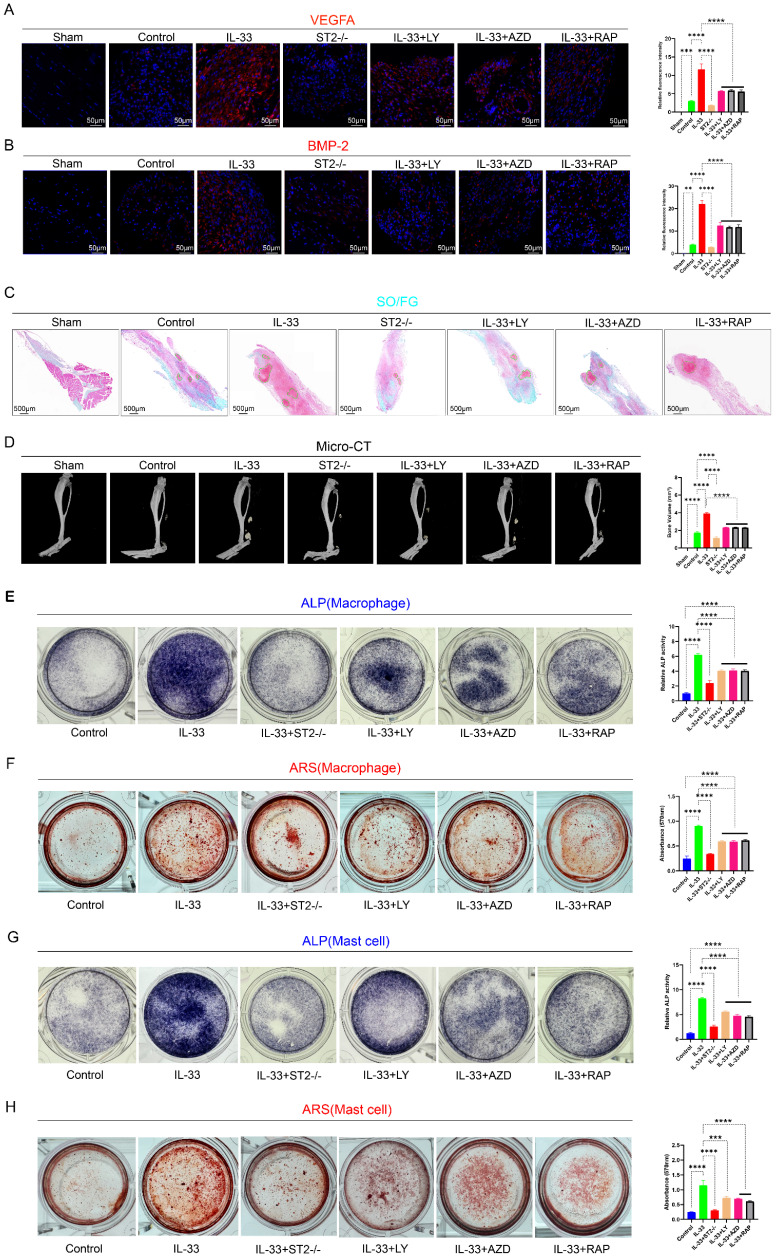
** IL-33/ST2-mediated M2 macrophage polarization and mast cell degranulation drive the formation of aberrant bone.** Establishment of models was mentioned before; during the osteogenic differentiation of TDSCs, the supernatants collected from BMDMs and BMMCs were respectively added to the TDSCs to investigate their effects on the differentiation process. **(A)** Immunofluorescence staining of VEGFA using injured tendon at 7 days after burn/tenotomy. **(B)** Immunofluorescence staining of BMP-2 using injured tendon at 7 days after burn/tenotomy. **(C)** SO/FG staining of tHO samples from mice at 3 weeks after burn/tenotomy. **(D)** Micro-CT scan results of tHO samples from mice at 10 weeks after burn/tenotomy. **(E, F)** ALP and ARS staining results in TDSCs from different groups treated with BMDM supernatants. **(G, H)** ALP and ARS staining results in TDSCs from different groups treated with BMMC supernatants. N=3/group, *P < 0.05, **P < 0.01, ***P < 0.001, ****P < 0.0001. All data are presented as mean ± SD.

Post-burn/tenotomy, the pathological process of aberrant bone formation encompasses three critical stages: the inflammatory stage (7 days post-injury), the chondrogenic stage (3 weeks post-injury), and the mature ectopic ossification stage (10 weeks post-injury). To elucidate the pivotal role and pathogenic effect of IL-33/ST2-mediated autophagy suppression in inflammatory cells on the pathogenesis of trauma-induced heterotopic ossification, we utilized multiple detection techniques to analyze injured tendons with six different treatments across three critical time points. Firstly, we utilized immunofluorescence staining to assess the expression levels of BMP-2 and VEGFA in tissues at 7 days post-model establishment. VEGFA enhances angiogenesis around the injured tissue, thereby supplying oxygen and nutrients to support heterotopic ossification. BMP-2, a pivotal driver of heterotopic ossification, triggers the differentiation of mesenchymal stem cells into osteoblasts, thus promoting the development of cartilage and bone tissue. M2 macrophages and mast cells are both capable of secreting these proteins, thereby activating downstream signaling pathways 42-44. The results demonstrated that, compared to the Sham group, the Control group exhibited elevated expression levels of VEGF and BMP-2, whereas the IL-33 group markedly upregulated protein expression. In contrast, the ST2-/- group showed slightly reduced expression levels compared to the control group due to the absence of the corresponding receptor. Autophagy rescue agents, specifically PI3K, AKT, and mTOR pathway inhibitors, effectively reversed the effects of IL-33 (Fig. 9A, B). Subsequently, we utilized Safranin O/Fast Green (SO/FG) staining for histological analysis of samples at 3 weeks post-injury and employed micro-CT for quantitative assessment of traumatic heterotopic ossification (tHO) formation at 10 weeks post-injury. The results of both demonstrated similar trends (Fig. 9C, D). The results demonstrated that IL-33 treatment markedly augmented the volume of traumatic heterotopic ossification (tHO) compared to the control and ST2-/- groups, whereas PI3K, AKT, and mTOR pathway inhibitors partially mitigated tHO formation. ST2-knockout mice displayed a significantly lesser tHO volume following trauma compared to Wild-type (Control group) mice (ST2-/- group). Interestingly, no statistically significant differences were observed in tHO volume among the three inhibitor groups.

To further elucidate the impact of IL-33/ST2-mediated inflammatory cell alterations on osteogenic differentiation of TDSCs, we performed *in vitro* osteogenic induction experiments. Supernatants were harvested from BMDMs and BMMCs subjected to different drug treatments for 8 hours. Subsequently, TDSCs were randomly allocated into two primary groups, each comprising six subgroups, and cultured in osteogenic induction medium to promote osteogenic differentiation. During this procedure, one group was supplemented with macrophage-derived supernatant, while the other received mast cell-derived supernatant. The results from Alkaline phosphatase (ALP) staining at 7 days and Alizarin Red S (ARS) staining at 21 days showed that both the supernatant derived from IL-33-treated macrophages and mast cells significantly enhanced the osteogenic differentiation capacity of TDSCs compared to the Control group. However, the osteogenic differentiation efficacy of the supernatant from three inhibitor groups was hampered, with no statistically significant differences observed among them (Fig. 9E, F for macrophages; Fig. 9G, H for mast cells).

Our multi-stage analysis reveals that IL-33/ST2 signaling orchestrates heterotopic ossification through autophagy-mediated enhancement of VEGF/BMP-2 secretion and subsequent TDSC osteogenic differentiation, providing a mechanistic link between early inflammation and late bone formation.

### BC hydrogel-sST2 Composite Scaffolds Reverse IL-33/ST2 axis-Driven Macrophage Polarization and Mast Cell Degranulation to Attenuate Post-Traumatic Heterotopic Ossification

#### Characterization of Composite Scaffolds

**Figure 10:**
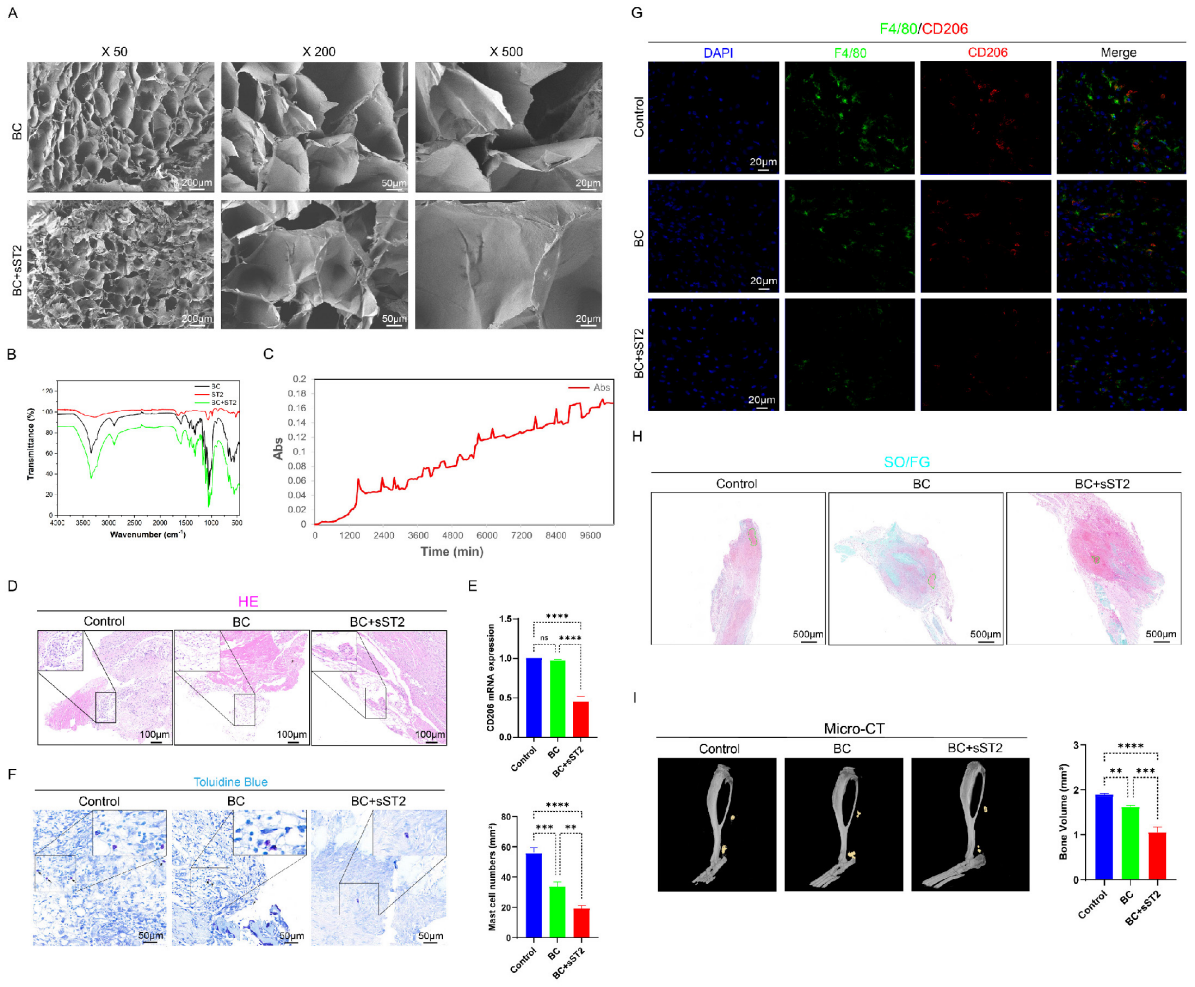
** Characterization of BC hydrogel-ST2 Composite Scaffolds and its Counteraction Against IL-33-Induced Inflammatory Storm and Heterotopic Ossification. (A)** SEM images of BC, BC+ST2 at varying magnifications, Scale bar, 2000 µm, 50 µm and 20 µm, respectively. **(B)** FTIR of the BC, BC+ST2. **(C)** Cumulative absorption values of ST2 release from BC+ST2 in normal saline. **(D)** H&E staining showing injured tissue cellularity at 7 days post-burn/tenotomy. **(E)** CD206 mRNA expression detected by qRT-PCR. **(F)** Toluidine blue (TB) staining showing mast cell accumulation and activation at 7 days post-burn/tenotomy. **(G)** Double fluorescence staining of F4/80 and CD206 at 7 days post-trauma. **(H)** SO/FG staining of tHO samples from mice at 3 weeks after burn/tenotomy. **(I)** Micro-CT scan results of tHO samples from mice at 10 weeks after burn/tenotomy. N=3/group, *P < 0.05, **P < 0.01, ***P < 0.001, ****P < 0.0001. All data are presented as mean ± SD.

The sST2-functionalized bacterial cellulose (BC) hydrogel composite scaffold was fabricated through physical entrapment, where sST2 molecules were immobilized within the nanofibrillar network via non-covalent interactions. Scanning electron microscopy (SEM) revealed that the pure freeze-dried bacterial cellulose (BC) hydrogel scaffold exhibits a porous nanofibrillar network structure with smooth inner walls. Following sST2 loading, the hydrogel's intrinsic porous architecture remained intact, with the emergence of aggregated sST2 particulate deposits on its surface. (Figure 10A) Fourier-transform infrared (FTIR) spectroscopy, employed for chemical bond analysis, demonstrated that sST2 incorporation generated vibrational signatures congruent with native BC functional groups, as evidenced by comparable characteristic band profiles in transmittance spectra. (Figure 10B) Subsequently, we conducted in vitro drug release assays of the composite scaffold. As depicted in Figure 10C, sST2 loaded within the bacterial cellulose (BC) hydrogel scaffold exhibited sustained release into normal saline medium, achieving near-complete release by day 7. This release profile was quantified through measurement of cumulative sST2 concentration in normal saline over time. (Figure 10C)

#### BC hydrogel-sST2 Composite Scaffolds Reverses IL-33-Driven Post-Traumatic Heterotopic Ossification In Vivo

As described in the Methods section, mice were divided into three groups: Control, BC group, and BC+sST2 group. Tissue harvesting was performed at 7 days (7D), 3 weeks (3W), and 10 weeks (10W) after establishing the burn/tenotomy model. Tissues collected at 7D post-injury were primarily analyzed for inflammatory cell changes. H&E staining demonstrated that compared to the Control group, application of BC hydrogel reduced inflammatory cell numbers in injured tissues. However, the BC+sST2 composite hydrogel exhibited a more pronounced effect, substantially decreasing inflammatory cell infiltration. (Fig. 10D) Toluidine Blue staining of injured tissues at 7D revealed that activated mast cells followed a descending order: Control group > BC group > BC+sST2 group, confirming that the composite scaffold significantly reversed IL-33-induced mast cell activation. (Fig. 10F) Concurrently, we quantified CD206 mRNA expression in 7D tissues via PCR to assess M2 macrophage populations. Notably, BC hydrogel alone failed to reduce CD206 mRNA expression, whereas the composite scaffold significantly decreased this value. (Fig. 10E) This was further confirmed by Immunofluorescence staining for M2 macrophage markers F4/80 and CD206 (Fig. 10G). Subsequent Safranin O/Fast Green staining of 3W tissues was employed to evaluate chondrogenesis. Compared to Controls, BC+sST2 composite scaffold application markedly reduced cartilage formation area at 3W post-injury, while BC hydrogel alone only moderately attenuated cartilage formation. (Fig. 10H) This consistent trend was similarly observed in micro-CT scanning of heterotopic bone at 10 weeks post-injury. Application of the composite scaffold significantly reduced the formation of heterotopic ossification, whereas the effect of BC hydrogel alone was less pronounced. (Fig. 10I)

Collectively, the BC+sST2 composite hydrogel demonstrates superior efficacy over BC alone in mitigating early inflammatory responses and subsequent heterotopic ossification progression, highlighting the therapeutic potential of targeting IL-33 signaling in HO intervention.

## Discussion

Heterotopic ossification (HO) is a pathological process characterized by the abnormal formation of lamellar bone in soft tissues such as muscle, tendon, and ligaments 1-2. It is generally believed that, under inductive factors and a permissive osteogenic microenvironment, osteoprogenitor cells such as tendon-derived stem cells (TDSCs) undergo aberrant differentiation. Thus, HO can be regarded as a stem cell disease—a lineage differentiation error driven by an abnormal microenvironment. This process involves disrupted normal soft tissue healing coupled with enhanced abnormal bone formation, ultimately leading to the replacement of normal tendon repair with aberrant osteogenic healing 45-46. Clinically, HO is frequently observed following severe trauma (e.g., fractures, joint replacements—particularly of the hip and elbow), a condition referred to as traumatic heterotopic ossification (tHO). Patients commonly present with symptoms including joint pain, swelling, and restricted mobility, which may progress to joint ankylosis in severe cases, significantly compromising quality of life 1-3. Surgical excision remains the primary treatment for mature tHO to restore joint function, although it is associated with a high risk of recurrence 47. To date, effective strategies for prevention and treatment remain limited in clinical practice. Thus, elucidating the molecular mechanisms underlying the pathogenesis of tHO and identifying novel targets for its prevention and treatment have become major research priorities.

Interleukin-33 (IL-33) functions as a damage-signaling cytokine that is passively released from the nuclei of necrotic structural cells to initiate immune responses via the ST2 receptor 48. Emerging evidence positions IL-33 as a master regulator spanning the continuum of tissue injury to repair. During the acute inflammatory phase, IL-33 acts as a critical alarmin that rapidly mobilizes the innate immune response. It activates ST2-expressing immune cells, orchestrating their recruitment to the injury site and stimulating the production of pro-inflammatory mediators such as IL-6 and CCL2. This cascade is essential for the initial clearance of cellular debris and pathogens 49-50. As inflammation resolves, IL-33 pivots to direct the repair process by enhancing fibroblast proliferation and collagen synthesis, thereby accelerating tissue regeneration 51. However, in the context of persistent injury, this reparative function may become dysregulated and excessively activated; chronic IL-33 signaling has been implicated in driving pathological fibrosis across various organs 52. This dual role is exemplified in liver injury models: following acute damage, IL-33 recruits eosinophils via a macrophage-dependent pathway, amplifying protective inflammation and supporting hepatocyte regeneration 17. Yet, when injury becomes chronic, the same signaling axis disrupts the equilibrium between inflammation and repair, ultimately promoting hepatic fibrosis 52. Notably, we detected overexpression of the IL-33/ST2 axis in human tHO samples compared to normal tendon tissue.

Specifically, trauma-induced heterotopic ossification (tHO) progresses through five distinct stages: the initial inflammatory storm phase, the mesenchymal cell recruitment and osteoprogenitor activation phase, the chondrogenesis phase, the cartilage mineralization and ossification phase, and the bone remodeling phase 53-56. Previous studies have indicated that the fate of mesenchymal stem cells (MSCs) can be determined as early as 3 days post-injury, highlighting the early inflammatory stage—characterized by a severe immune cell response—as a key therapeutic window in HO formation 57. In this study, we established a burn/tenotomy model to replicate post-traumatic HO formation, harvesting tissue samples at three critical time points: the early inflammatory phase (3 and 7 days post-injury), the chondrogenesis phase (3 weeks post-injury), and the HO maturation phase (10 weeks post-injury). Surprisingly, our experiment revealed a marked upregulation of IL-33 expression during the early inflammatory phase. We also observed high ST2 expression on CD206⁺ macrophages and tryptase⁺ mast cells. Based on this observation, we hypothesized that IL-33 promotes HO formation through a cascade initiated by its action on ST2-expressing inflammatory cells, which activates downstream signaling pathways.

It is well-documented that the IL-33/ST2 axis can target both mast cells and macrophages in various other pathological conditions. Within the process of asthma pathogenesis, the IL-33/ST2 axis is known to drive mast cell activation, resulting in the release of pro-allergic cytokines and chemokines that exacerbate airway hyperresponsiveness 13,58-59. Numerous studies have reported the accumulation of mast cells around injured tendons during the early post-traumatic period, where they contribute to the inflammatory response and HO development through degranulation. Moreover, the IL-33/ST2 axis is involved in M2 macrophage polarization. As mentioned before, M2 macrophage are widely recognized for their tissue-reparative functions, secreting cytokines that facilitate tissue regeneration, remodeling, angiogenesis, and systemic homeostasis. Jiang et al. demonstrated that Celastrol attenuates brain injury following acute ischemic stroke by inducing IL-33/ST2-mediated M2 polarization of microglia /macrophages 60. Xu et al. revealed that M2 macrophages promote tendon healing by stimulating fibroblasts to secrete collagen and enhancing the regeneration of endothelial cells 61. Our previous research also confirmed that M2 macrophages can contribute to the formation of ectopic ossification through the secretion of TGF-β and VEGF 12.

Consistent with these findings, our experimental results showed an increased presence of degranulated mast cells in samples locally injected with IL-33 at the tendon site. Similarly, IL-33 administration enhanced M2 macrophage polarization around the injured tendon. *In vitro* experiment using further confirmed that IL-33 plays a role in mast cells degranulation and M2 macrophage polarization. We found that the cytokines such as VEGFA and BMP-2, which could induce the osteogenic differentiation of TDSCs, sharply increased in the IL-33 group while decreased in the ST2-/- group. Moreover, TDSCs exposed to supernatants from WT BMDMs and WT BMMCs treated with IL-33 demonstrated significantly enhanced osteogenic differentiation potential compared to controls and ST2-/- cells, as evidenced by increased ALP activity. Collectively, these findings confirmed that IL-33/ST2 axis induced tHO by driving the degranulation of mast cells and M2 macrophage polarization.

To delineate the downstream molecular mechanisms, we performed proteomic sequencing on animal models and screened for highly significant autophagy-related pathways from a broad range of enrichment analyses. Two autophagy-related pathways—Lysosome Vesicle Biogenesis and COPII-mediated Vesicle Transport—were identified among the top 20 most significant Reactome pathways in our dataset. A correlated upregulation of both pathways was observed in the ST2-knockout (KOE) group compared to the wild-type (EG) control group. Mechanistically, COPII-mediated vesicle transport provides the essential membrane materials and key proteins for autophagosome nucleation and elongation 62. Its upregulation indicates that cells are actively gathering "building materials" to initiate autophagy. Conversely, lysosome vesicle biogenesis is indispensable for ensuring unimpeded autophagic flux. Its upregulation signifies that cells are preparing the "degradative capacity" necessary to execute the final steps of autophagy 63. These findings strongly suggest that the autophagy process is likely significantly activated in the ST2-deficient mouse model. The cells are not only allocating resources for the initiation phase but are also preparing for the degradation phase, demonstrating a coherent and orchestrated activation of the complete autophagic cascade. We subsequently employed GSEA analysis, which further corroborated the upregulation of autophagy in the ST2-deficient mouse model.

Autophagy is a vital process in eukaryotic cells that maintains intracellular homeostasis by degrading cytoplasmic proteins and damaged organelles via lysosome-mediated pathways. Regulated by autophagy-related genes, it begins with the formation of a double-membrane phagophore that engulfs cytoplasmic material. The phagophore matures into an autolysosome through fusion with a lysosome, where lysosomal enzymes break down its contents. The resulting metabolites, such as amino acids and fatty acids, are recycled for cellular use 19,64-65. Autophagy is widely recognized as a critical regulator of inflammatory responses 66-68. Under physiological conditions, moderate autophagic activity serves to restrain excessive inflammation and maintain immune homeostasis. Conversely, impairment of autophagic flux can lead to dysregulated inflammatory activation and promote chronic inflammatory states. Notably, under specific pathological contexts, hyperactivated autophagy may also exacerbate cellular stress and amplify inflammatory signaling, revealing the dual nature of autophagy in immune regulation. This complexity is particularly evident in the ongoing debate regarding the relationship between autophagy and mast cell degranulation. Supporting one perspective, Nian et al. demonstrated that in allergic rhinitis, epithelial-derived IL-33 facilitates mast cell degranulation through autophagy suppression 69. This aligns with our findings, where IL-33 not only promotes mast cell recruitment to injury sites during early inflammation but also inhibits autophagic initiation in mast cells via ST2 signaling, thereby enhancing degranulation. In contrast, Li et al. reported that ORMDL3 overexpression induces degranulation in a manner reversible by autophagy inhibition 70. These apparently contradictory observations may stem from differences in the stimuli (IL-33 versus ORMDL3 overexpression) and their corresponding inflammatory intensities, highlighting the context-dependent role of autophagy in mast cell biology.

Similarly, autophagy deficiency has been implicated in macrophage polarization. Liu et al. observed in an obesity model that defective macrophage autophagy promoted M1 polarization, exacerbating immune-mediated liver injury 71. In a related vein, Wei et al. found that PD-L1 induces M2 polarization through ERK/AKT/mTOR activation 72—a pathway known to suppress autophagy, though the causal link was not explicitly investigated. In our burn/tenotomy model, we further demonstrated that the IL-33/ST2 axis drives M2 polarization of macrophages in an autophagy-dependent manner, wherein autophagy inhibition appears to be a key mechanistic step.

Mechanistic target of rapamycin(mTOR) is known for a central regulator of cell growth, metabolism, and autophagy. Its inhibitory effect on autophagy is primarily mediated through the mTORC1 complex via two main mechanisms: 1. mTORC1 phosphorylates ULK1, preventing its interaction with and activation by AMPK, thereby blocking the initiation of autophagosome formation. 2.mTORC1 also phosphorylates the transcription factor TFEB, retaining it in the cytoplasm and preventing its nuclear translocation, which is necessary for the transcription of autophagy-related genes 73-74. Studies show that the IL-33/ST2 axis drives the activation and proliferation of immune cells by activating the PI3K/AKT pathway, thereby playing a role in various diseases such as autoimmune disorders, allergic reactions, and tumorigenesis 75-76. Specifically, Zhu et al. demonstrated that in gastric carcinogenesis, IL-33, via the ST2 receptor, activates the TRAF6/PI3K/Akt/NF-κB pathway, thereby triggering the subsequent VEGFA-induced angiogenesis, which provides blood supply and nutrients for tumor formation 77. Song et al. identified that the IL-33/ST2 axis contributes to systemic lupus erythematosus (SLE) progression through a mechanism driven by the Ro60 upregulation in keratinocytes, which is activated via the PI3K/Akt and SOX17 pathways and subsequently recruits macrophages 78. More intuitive evidence reveals that IL-33/ST2 signaling contributes to the activation of the PI3K/mTOR pathway and the subsequent suppression of autophagy. This, in turn, induces mast cell degranulation, ultimately exacerbating allergic rhinitis 69. Consistently, our experiment found that IL-33/ST2 axis can increase the level of p-AKT, thereby further activating mTOR and exerting an inhibitory effect on autophagy in BMDMs and BMMCs. The subsequent autophagy rescue experiments using PI3K, AKT, MTOR inhibitors further validated that IL-33/ST2 axis drove the inhibition of autophagy in mast cells and macrophages through a PI3K/AKT/mTOR- related way. Moreover, treatment with these inhibitors not only reversed the IL-33-induced mast cell degranulation and M2 macrophage polarization but also attenuated tHO formation. Collectively, we found that PI3K/AKT/mTOR-induced autophagy suppression in mast cells and macrophages caused by IL-33/ST2 axis drove their functional stage changes and finally leading to the formation of tHO.

The soluble ST2 receptor (sST2), a member of the IL-1 receptor family, is a secreted isoform generated through alternative splicing of the IL1RL1 gene. Unlike the transmembrane ST2L receptor, sST2 lacks both the transmembrane domain and intracellular TIR domain. Upon binding IL-33, the resulting sST2/IL-33 complex undergoes lysosomal degradation rather than initiating downstream signal transduction 79-81. Consequently, sST2 functions as a competitive inhibitor of the IL-33 signaling pathway. This molecular mechanism enables sST2 to suppress IL-33/ST2 axis-mediated M2 polarization of macrophages and attenuate mast cell degranulation. However, exogenously administered sST2 is highly susceptible to degradation in biological systems 82. To overcome this limitation, we leveraged the exceptional drug-loading capacity of bacterial cellulose (BC) hydrogel to achieve controlled release of sST2. The composite scaffold we synthesized demonstrates high biocompatibility and ideal controlled drug-release functionality. *In vitro* release experiments confirmed favorable performance of physically entrapped sST2: intermittent release initiated immediately upon testing commencement and persisted through day 7. For *in vivo* evaluation, mice were randomly divided into three groups: a control group, a group receiving pure bacterial cellulose (BC) hydrogel implantation, and a group implanted with sST2-loaded BC hydrogel. Results indicated that pure BC hydrogel exerted modest anti-inflammatory and anti-heterotopic ossification effects. Following drug loading, sustained sST2 release from the composite scaffold competitively bound trauma-induced IL-33 at the injury site. This significantly reversed M2 polarization of ST2 receptor-expressing macrophages and suppressed mast cell activation, ultimately reducing cartilage formation and heterotopic bone tissue development. Through this sustained-release system, we further validated our prior findings regarding IL-33's molecular mechanism in promoting heterotopic ossification via ST2 receptor signaling, thereby establishing a novel prevention strategy.

Our study has certain limitations. First, in the autophagy rescue experiments, we only used wild-type mouse tendon samples and cells, and the impact of the inhibitor on autophagy trends in ST2-knockout mice was not performed. What's more, in the *in vitro* osteogenesis induction experiment with TDSCs, we directly used the supernatant from macrophages and mast cells that stimulated with recombinant IL-33. Theoretically, the supernatant still contained residual IL-33, and whether this IL-33 can also inhibit autophagy and affect the osteogenesis process remains to be further studied.

Additionally, regarding bacterial cellulose (BC) hydrogel, it still presents certain limitations. First, its dense nanofibrillar network may result in insufficient pore size, thereby restricting the loading capacity and efficiency for sST2 and consequently compromising its efficacy in counteracting IL-33-induced heterotopic ossification and subsequent clinical translation. In subsequent studies, we plan to integrate chitosan into the scaffold to modify its pore structure, thereby enhancing the sST2 loading capacity and further evaluating its therapeutic efficacy. On the other hand, our experimental findings confirm that its sustained-release performance requires further improvement. The BC-sST2 delivery system engineered in our study currently sustains controlled sST2 release for up to 7 days, which can't fully cover the inflammatory stage of HO (usually lasts for 14 days). This phenomenon may be attributed to the current reliance on solely physical interactions for immobilizing sST2 within the bacterial cellulose hydrogel matrix. To extend release kinetics to ≥14 days, we propose synthesizing next-generation scaffolds through covalent conjugation strategies. This advancement promises enhanced prophylactic efficacy against heterotopic ossification. On the other hand, we plan to synthesize chiral bacterial cellulose hydrogel-based drug delivery scaffolds to achieve improved drug loading capacity and sustained-release profiles. Finally, it should be noted that our study revealed that BC hydrogel itself exhibits certain anti-heterotopic ossification properties due to its inherent anti-inflammatory characteristics. While the incorporation of sST2 enhanced this anti-HO effect, whether these two components exert synergistic anti-ossification activity requires further investigation.

In conclusion, this study establishes IL-33 as a critical alarmin mediating trauma-induced heterotopic ossification (tHO) through a defined molecular cascade. Mechanistically, IL-33 binding to ST2 receptors on mast cells and macrophages suppresses autophagy, leading to mast cell degranulation and M2 macrophage polarization. These activated immune cells secrete cytokines that establish a pro-osteogenic microenvironment, promoting tendon-derived stem cell (TDSC) differentiation and driving aberrant bone formation. Based on these insights, we developed a bacterial cellulose (BC) hydrogel delivery system for sustained sST2 release. This biomaterial-based strategy functions as a decoy receptor that competitively inhibits IL-33 signaling, subsequently restoring autophagy and attenuating heterotopic bone formation. Our findings not only elucidate the "alarmin-autophagy-immune" axis in tHO pathogenesis but also propose multiple therapeutic strategies: suppressing IL-33 release, blocking ST2 receptor signaling, restoring autophagy via pathway inhibitors, or implementing sST2 sustained-release systems. This work significantly advances our understanding of inflammatory mechanisms in ectopic ossification and provides a translational framework for tHO prevention and treatment.

## Supplementary Material

Supplementary figures and tables.

## Data Availability

Date and material will be made available on reasonable request.
