# Supplementary material for: The IL-33/ST2 Axis Promotes Traumatic Heterotopic Ossification by Driving Macrophage and Mast Cell-Mediated Inflammation via Autophagy Defects
Source: Int J Biol Sci. 2026 Jan 1;22(1):60–85. doi: 10.7150/ijbs.122706 (PMC12681708; doi:10.7150/ijbs.122706)
Supplement: Supplementary file 1 — Supplementary figures and tables. [file ijbsv22p0060s1.pdf]

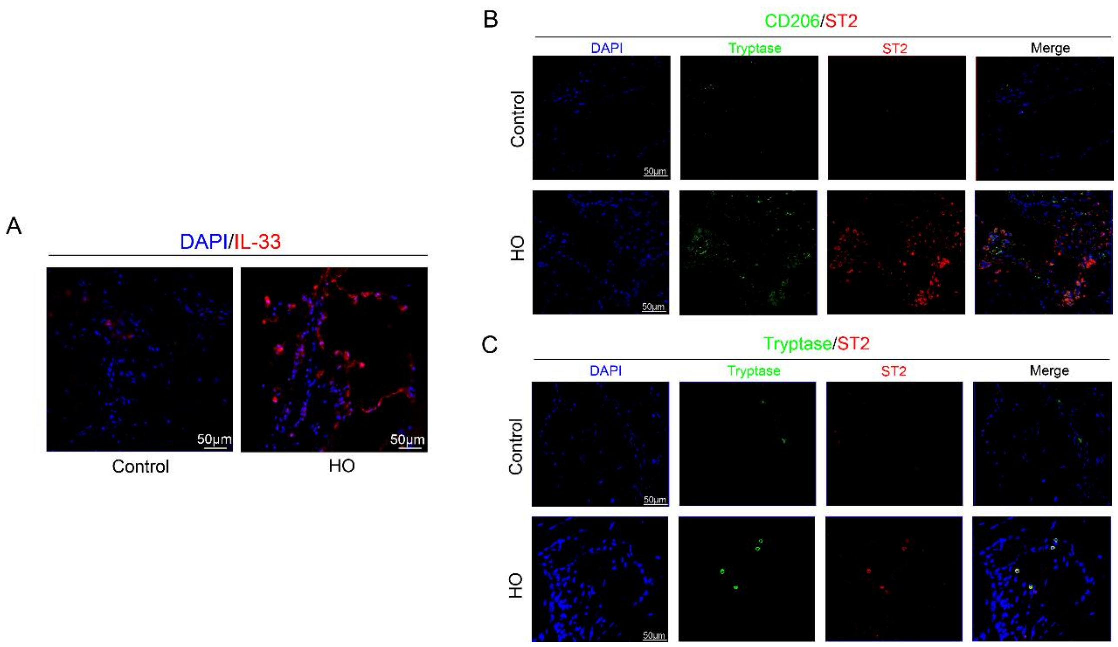

Supplement Figure 1 | Overexpression of the IL-33/ST2 axis in human HO samples. Immunofluorescence of IL-33. (B) Double fluorescence staining of ST2 and CD206 or Tryptase. N=6/group, *$P < 0.05$, **$P < 0.01$, ***$P < 0.001$, ****$P < 0.0001$. All data are presented as mean ± SD.

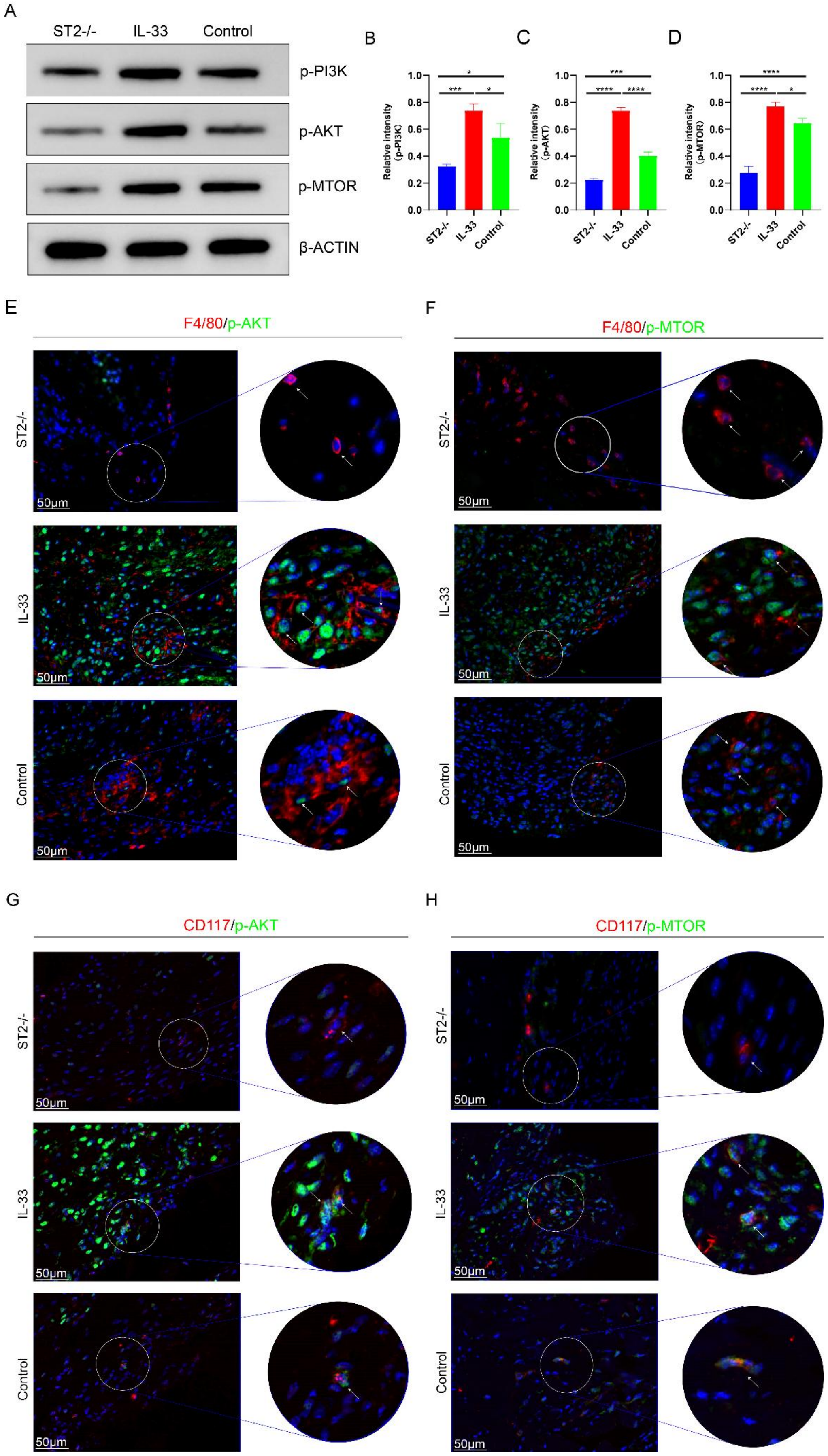
A
ST2-/-
IL-33
Control
p-PI3K
p-AKT
p-MTOR
β-ACTIN
B
C
D
Relative intensity (p-PI3K)
Relative intensity (p-AKT)
Relative intensity (p-MTOR)
1.0
0.8
0.6
0.4
0.2
0.0
*
***
****
E
F4/80/p-AKT
F
F4/80/p-MTOR
G
CD117/p-AKT
H
CD117/p-MTOR
50μm

Supplement Figure 2 | PI3K/AKT/MTOR Axis Were Actived in A IL-33/ST2-Dependent Way After Burn/Tenotomy

(A, B, C, D) Western blot analysis and quantification of p-PI3K, p-AKT, and p-mTOR using injured tissue at 7 days post-trauma. (E,F)Double fluorescence staining of F4/80 and p-AKT or p-mTOR. (G, H) Double fluorescence staining of CD117 and p-AKT or p-mTOR. N=3/group, *P < 0.05, **P < 0.01, ***P < 0.001, ****P < 0.0001. All data are presented as mean ± SD.

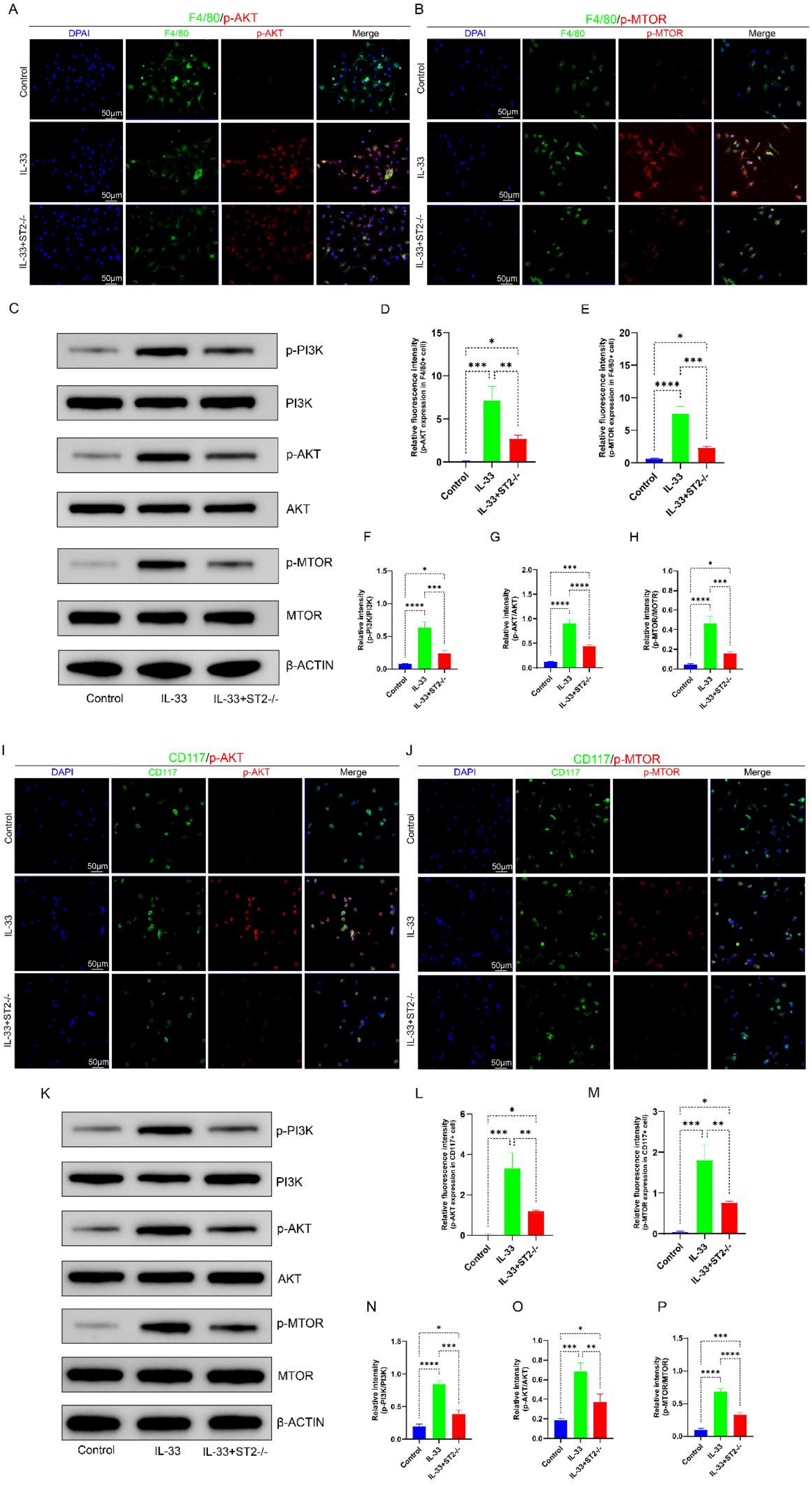
A
F4/80/p-AKT
DPAI
F4/80
p-AKT
Merge
Control
IL-33
IL-33+ST2-/-
50μm
B
F4/80/p-MTOR
DPAI
F4/80
p-MTOR
Merge
C
p-PI3K
PI3K
p-AKT
AKT
p-MTOR
MTOR
β-ACTIN
Control
IL-33
IL-33+ST2-/-
D
Relative fluorescence intensity
(p-AKT expression in F4/80+ cell)
E
Relative fluorescence intensity
(p-MTOR expression in F4/80+ cell)
F
Relative intensity
(p-PI3K/PI3K)
G
Relative intensity
(p-AKT/AKT)
H
Relative intensity
(p-MTOR/MOTR)
I
CD117/p-AKT
DAPI
CD117
p-AKT
Merge
J
CD117/p-MTOR
DAPI
CD117
p-MTOR
Merge
K
p-PI3K
PI3K
p-AKT
AKT
p-MTOR
MTOR
β-ACTIN
Control
IL-33
IL-33+ST2-/-
L
Relative fluorescence intensity
(p-AKT expression in CD117+ cell)
M
Relative fluorescence intensity
(p-MTOR expression in CD117+ cell)
N
Relative intensity
(p-PI3K/PI3K)
O
Relative intensity
(p-AKT/AKT)
P
Relative intensity
(p-MTOR/MTOR)

Supplement Figure 3 | IL-33/ST2 induces autophagy inhibition via the PI3K/AKT/mTOR pathway

BMDMs and BMMCs were treated as described in Figures 4 and 5.

(A, B, D, E) Double fluorescence staining of F4/80 and p-AKT or p-mTOR in BMDMs. (C, F, G, H) Western blot analysis and quantification of p-PI3K, p-AKT, and p-mTOR in BMDMs. (I, J, L, M) Double fluorescence staining of CD117 and p-AKT or p-mTOR in BMMCs. (K, N, O, P) Western blot analysis and quantification of p-PI3K, p-AKT, and p-mTOR in BMMCs. N=3/group, *$P < 0.05$, **$P < 0.01$, ***$P < 0.001$, ****$P < 0.0001$. All data are presented as mean ± SD.

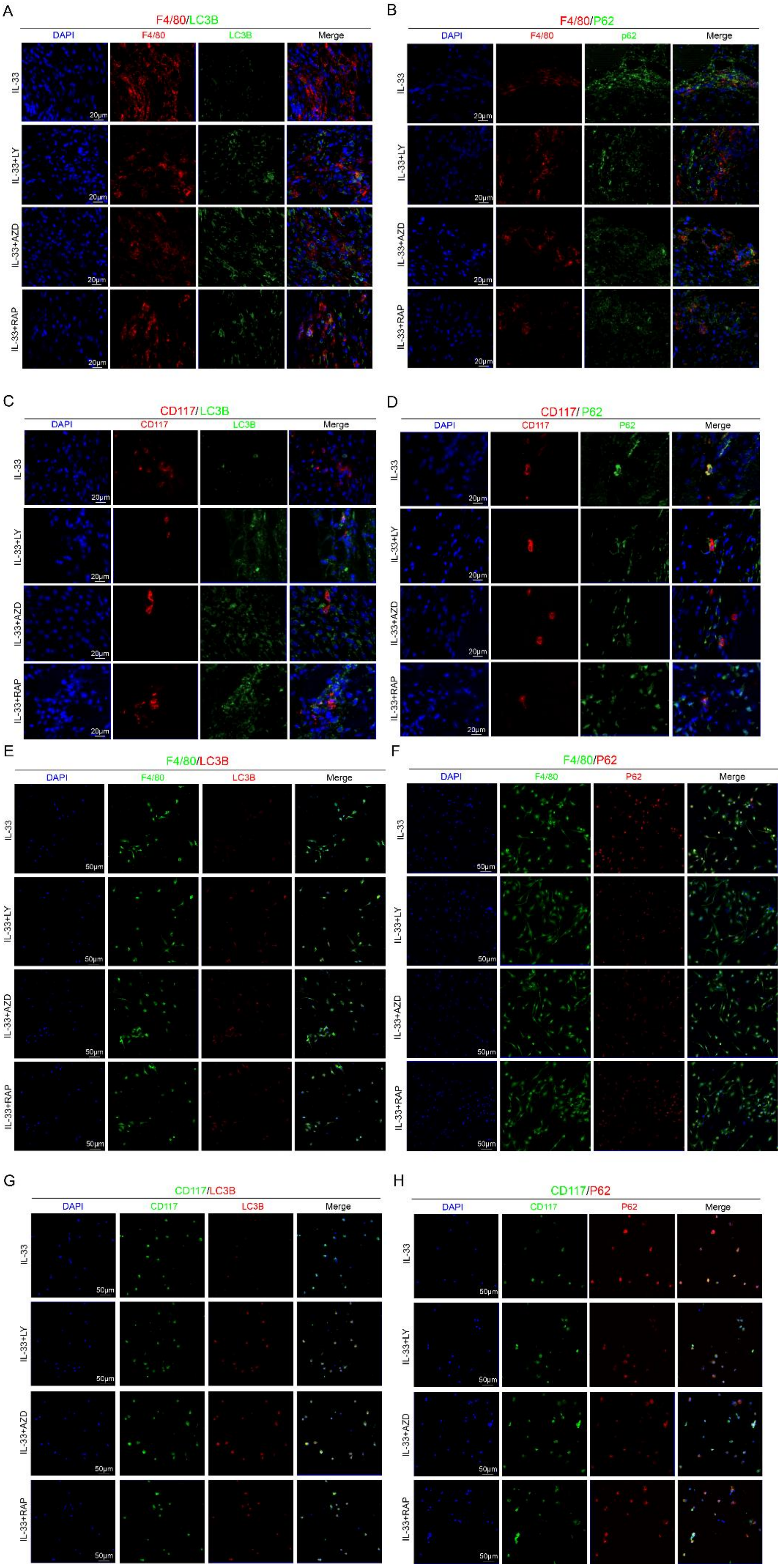
A
F4/80/LC3B
DAPI
F4/80
LC3B
Merge
IL-33
IL-33+LY
IL-33+AZD
IL-33+RAP
20μm
B
F4/80/P62
DAPI
F4/80
p62
Merge
IL-33
IL-33+LY
IL-33+AZD
IL-33+RAP
20μm
C
CD117/LC3B
DAPI
CD117
LC3B
Merge
IL-33
IL-33+LY
IL-33+AZD
IL-33+RAP
20μm
D
CD117/ P62
DAPI
CD117
P62
Merge
IL-33
IL-33+LY
IL-33+AZD
IL-33+RAP
20μm
E
F4/80/LC3B
DAPI
F4/80
LC3B
Merge
IL-33
IL-33+LY
IL-33+AZD
IL-33+RAP
50μm
F
F4/80/P62
DAPI
F4/80
P62
Merge
IL-33
IL-33+LY
IL-33+AZD
IL-33+RAP
50μm
G
CD117/LC3B
DAPI
CD117
LC3B
Merge
IL-33
IL-33+LY
IL-33+AZD
IL-33+RAP
50μm
H
CD117/P62
DAPI
CD117
P62
Merge
IL-33
IL-33+LY
IL-33+AZD
IL-33+RAP
50μm

Supplement Figure 4 | Impaired Autophagy Can Be Restored by Inhibiting the PI3K/AKT/mTOR Pathway

(A, B) Double fluorescence staining of F4/80 and LC3B or p62 in tissue samples.(C,D)Double fluorescence staining of CD117 and LC3B or p62 in tissue samples.(E, F) Double fluorescence staining of F4/80 and LC3B or p62 in BMDMs .(G,H)Double fluorescence staining of CD117 and LC3B or p62 in BMMCs. N=3/group, *P < 0.05, **P < 0.01, ***P < 0.001, ****P < 0.0001.All data are presented as mean ± SD.

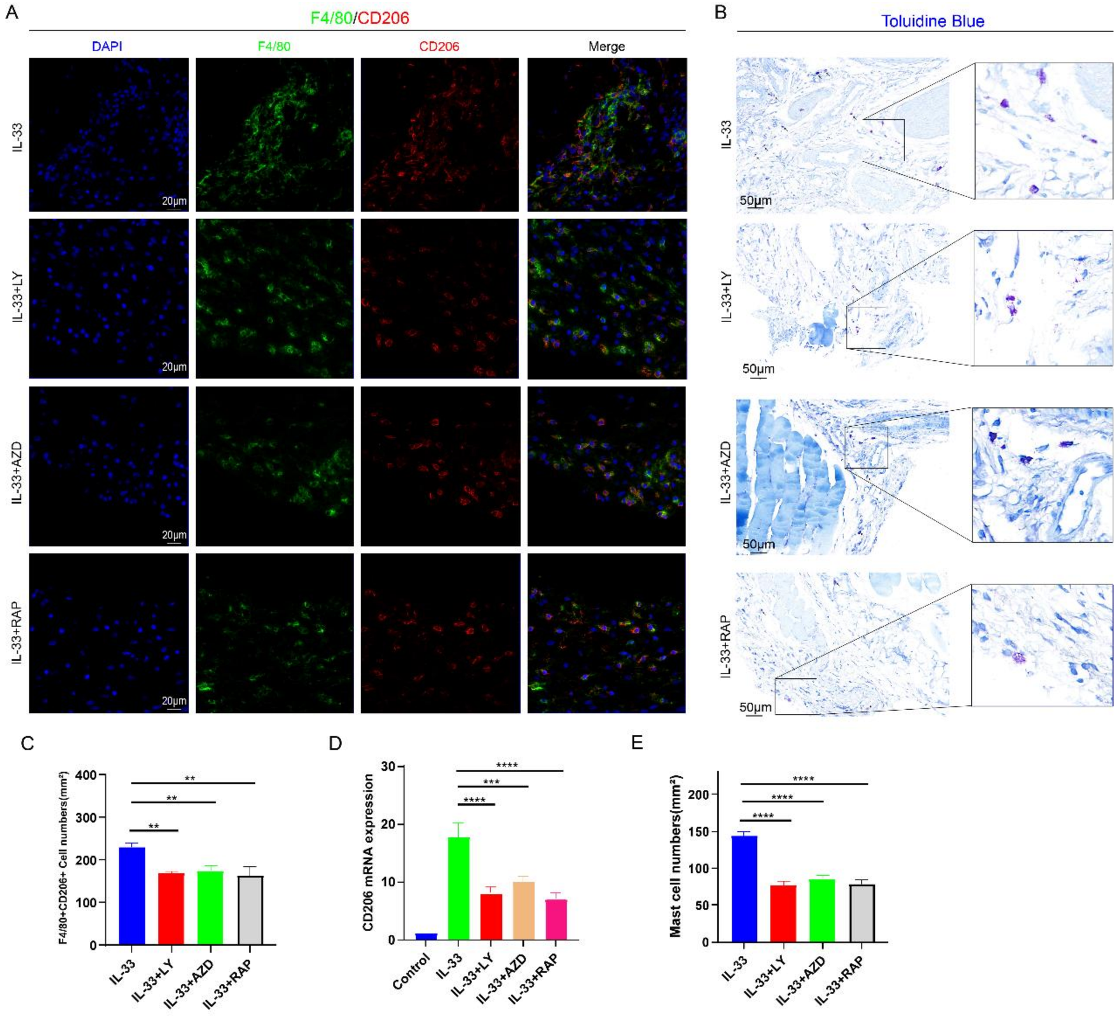

Supplement Figure 5 | Role of IL-33-Mediated Autophagy Suppression in Regulating M2 Macrophage Polarization and Mast Cell Degranulation

(A) Double fluorescence staining of F4/80 and CD206 at 7 days post-trauma. (B, E) Toluidine blue (TB) staining showing mast cell accumulation and activation at 7 days post-burn/tenotomy. (C) Quantification for F4/80 and CD206 double-positive cells reveled by fluorescence staining. (D)CD206 mRNA expression by qRT-PCR.N=3/group, *P < 0.05, **P < 0.01, ***P < 0.001, ****P < 0.0001.All data are presented as mean ± SD.

Table S1. Antibodies used in this study

Antibodies	Supplier	Catalog	Application
IL-33	Proteintech	12372-1-AP	IF
ST2	Proteintech	11920-1-AP	IF
P-PI3K	Bioss	bs-6417R-100	WB
P-AKT	Servicebio	GB150002-100	WB,IF
P-MTOR	Servicebio	GB114489-100	WB,IF
PI3K	Servicebio	GB11525-100	WB
AKT	Servicebio	GB15689-100	WB
MTOR	Servicebio	GB11405-100	WB
β-ACTIN	Servicebio	GB15001-100	WB
ARG-1	Servicebio	GB115724-100	WB
F4/80	Cell signaling technology	70076	IF
CD206	Proteintech	18704-1-AP	IF
CD117	Proteintech	18696-1-AP	IF
CD63	Abcam	ab217345	IF
Avidin	Bioss	bs-0312R-1mg	IF
LC3B	Proteintech	18725-1-AP	IF
P62	Proteintech	18420-1-AP	IF
VEGFA	Servicebio	GB15165-100	IF
BMP-2	Servicebio	GB15252-100	IF

Table S2. Primer sequence used for genotyping ST2 knockout mice

PCR No.	Primer No.	Primer Name	Sequence	Band Size
PCR①	F1	JS00407-IL1RL1--5wt-tF2	AAACGTGTAGATGCCAGAGCCTG	WT:1627bp KO: 302bp
	R1	JS00407-IL1RL1--3wt-tR2	CCTGCTTTGAAAACACTGATGATG	
PCR②	F2	JS10407-IL1RL1-wt-tF2	GATGTACTCGACAGTACGTGGATC	WT:554bp KO:0bp
	R2	JS10407-IL1RL1-wt-tR2	TTCTTAAACCTATGCTTCATGGC	

Table S3. Primers used for qRT-PCR

Genes	Forward	Reverse
Murine IL-33	ATTTCTGCGTCTGTTGACAC	CACCGTCGCCTGATTGACT
Murine ST2	CTCCAAGAGCCAAGGTTCAGG	CAAAGCAAGCTGAACAGGCAA
Murine CD206	TACTTGGACGGATAGATGGAGG	CATAGAAAGGAATCCACGCAGT
Murine GAPDH	CCTCGTCCCGTAGACAAAATG	TGAGGTCAATGAAGGGGTCGT
